# Transcriptome-Wide Association Study Provides Insights Into the Genetic Component of Gene Expression in Anxiety

**DOI:** 10.3389/fgene.2021.740134

**Published:** 2021-09-28

**Authors:** Xi Su, Wenqiang Li, Luxian Lv, Xiaoyan Li, Jinfeng Yang, Xiong-Jian Luo, Jiewei Liu

**Affiliations:** ^1^ Henan Mental Hospital, The Second Affiliated Hospital of Xinxiang Medical University, Xinxiang, China; ^2^ Henan Key Lab of Biological Psychiatry, International Joint Research Laboratory for Psychiatry and Neuroscience of Henan, Xinxiang Medical University, Xinxiang, China; ^3^ Key Laboratory of Animal Models and Human Disease Mechanisms of the Chinese Academy of Sciences and Yunnan Province, Kunming Institute of Zoology, Chinese Academy of Sciences, Kunming, China; ^4^ Kunming College of Life Science, University of Chinese Academy of Sciences, Kunming, China; ^5^ Center for Excellence in Animal Evolution and Genetics, Chinese Academy of Sciences, Kunming, China; ^6^ KIZ-CUHK Joint Laboratory of Bioresources and Molecular Research in Common Diseases, Kunming Institute of Zoology, Chinese Academy of Sciences, Kunming, China

**Keywords:** TWAS, GWAS, anxiety, PsychENCODE, GTEx

## Abstract

Anxiety disorders are common mental disorders that often result in disability. Recently, large-scale genome-wide association studies (GWASs) have identified several novel risk variants and loci for anxiety disorders (or anxiety traits). Nevertheless, how the reported risk variants confer risk of anxiety remains unknown. To identify genes whose *cis*-regulated expression levels are associated with risk of anxiety traits, we conducted a transcriptome-wide association study (TWAS) by integrating genome-wide associations from a large-scale GWAS (*N* = 175,163) (which evaluated anxiety traits based on Generalized Anxiety Disorder 2-item scale (GAD-2) score) and brain expression quantitative trait loci (eQTL) data (from the PsychENCODE and GTEx). We identified 19 and 17 transcriptome-wide significant (TWS) genes in the PsychENCODE and GTEx, respectively. Intriguingly, 10 genes showed significant associations with anxiety in both datasets, strongly suggesting that genetic risk variants may confer risk of anxiety traits by regulating the expression of these genes. Top TWS genes included *RNF123*, *KANSL1-AS1, GLYCTK, CRHR1, DND1P1, MAPT* and *ARHGAP27.* Of note, 25 TWS genes were not implicated in the original GWAS. Our TWAS identified 26 risk genes whose *cis*-regulated expression were significantly associated with anxiety, providing important insights into the genetic component of gene expression in anxiety disorders/traits and new clues for future drug development.

## Introduction

Anxiety disorders are common mental disorders (Huang et al., 2019) and a leading cause of disability (GBD 2017 Disease and Injury Incidence and Prevalence Collaborators, 2018). As an adaptive behavioral response, anxiety is usually a normal emotional experience in daily life. However, individuals affected by anxiety disorders frequently have feelings of excessive, intense, and persistent worry and fear about the actual or anticipated event. If the degree of anxiety exceeds a certain range and the abnormal feeling of fear and worry lasts for a long time, normal anxiety experiences may turn into anxiety disorders. Anxiety disorders are highly prevalent mental disorders (with a lifetime prevalence rate of over 20% (Kessler et al., 2012)) that bring huge economic burdens to the world (Baxter et al., 2014). The etiology of anxiety disorders remains poorly understood. However, it is found that genetic factors play pivotal roles. The heritability of anxiety disorders were estimated to range from 20 to 60% (Polderman et al., 2015; Craske et al., 2017), indicating substantial genetic components of anxiety disorders. To uncover the genetic basis of anxiety disorders/traits, several genetic studies have been performed (Sipilä et al., 2010; Stein et al., 2011; Nivard et al., 2014). Nevertheless, large-scale GWASs of anxiety disorders/traits have not emerged until recently (Otowa et al., 2016; Meier et al., 2019; Levey et al., 2020; Purves et al., 2020). For example, by using the UK biobank sample, Purves *et al.* identified five genome-wide significant (GWS) risk loci for anxiety disorder (Purves et al., 2020). In addition, Meier *et al.* reported that *PDE4B* is associated with anxiety and stress-related diagnoses in the Danish population (Meier et al., 2019). Otowa *et al.* also found that a noncoding RNA region located in 3q12.3 is associated with anxiety disorder (Otowa et al., 2016). Notably, a recent large-scale GWAS (*N* = 199,611) conducted by Levey *et al.* (i.e., the Million Veteran Program, MVP) reported six GWS loci for Generalized Anxiety Disorder 2-item scale (GAD-2) GWAS (Levey et al., 2020). There are also other GWASs for specific anxiety disorders/traits, such as Generalised Anxiety Disorder (Davies et al., 2015; Dunn et al., 2017)^,^ panic disorder (Otowa et al., 2009; Erhardt et al., 2011; Otowa et al., 2012), phobic anxiety (Walter et al., 2013), and social anxiety (Stein et al., 2017). Although these GWASs have identified several risk variants and loci for anxiety disorders/traits, the number of reported anxiety disorders/traits risk loci remains small compared with other psychiatric disorders (including schizophrenia (Schizophrenia Working Group of the Psychiatric Genomics Consortium, 2014; Pardiñas et al., 2018; Lam et al., 2019), depression (Wray et al., 2018), and bipolar disorder (Stahl et al., 2019)).

To date, most of the risk variants reported by anxiety disorders/traits GWASs are located in noncoding regions (Otowa et al., 2016; Meier et al., 2019; Levey et al., 2020; Purves et al., 2020), suggesting that these genetic variations confer risk of anxiety disorders/traits by modulating gene expression. However, we currently know little about the target genes regulated by these reported risk variants. In addition, the joint effects of the reported anxiety disorders risk loci on gene expression across human brain tissues remain elusive. The emergence of TWAS (Gusev et al., 2016) provides an opportunity to infer genes whose *cis*-regulated expression may be associated with diseases and traits. TWAS first leverages genotype and expression data from an external reference panel to identify the associations between genetic variations and gene expression. Then it identifies genes whose cis-regulated expression are associated with diseases or phenotypes by integrating gene expression data (i.e., external reference panel) with genome-wide associations from large-scale GWASs. TWASs have been widely used to identify risk genes for psychiatric disorders and neurodegenerative disease, including schizophrenia (Gandal et al., 2018), depression (Dall'Aglio et al., 2020), Parkinson’s disease (Li et al., 2019), and attention deficit hyperactivity disorder (Liao et al., 2019). Moreover, TWASs have identified multiple novel risk genes that were not implicated by the original GWASs (Liao et al., 2019).

In this study, we performed a TWAS (using FUSION software (Gusev et al., 2016)) on anxiety traits by integrating genome-wide associations from a recent large-scale GAD-2 score GWAS of anxiety disorder (Levey et al., 2020) (i.e., the Million Veteran Program) and gene expression data from multiple brain tissues. We identified 26 TWS genes, of which 19 were from the PsychENCODE gene expression reference and 17 were from GTEx. It is noteworthy that 10 genes (*KANSL1-AS1*, *CRHR1, CRHR1-IT1, SPPL2C, RP11-707O23.5, RP11-259G18.1, MAPT-AS1, LRRC37A4P, PLEKHM1,* and *DND1P1*) showed TWS in both datasets (i.e., PsychENCODE and GTEx), implying these genes are promising candidate genes for anxiety. Moreover, we noticed that some novel risk genes identified by our TWAS were not implicated in the original GWAS. Our study identifies genes whose expression change may confer risk of anxiety disorders/traits, shading lights on the genetic component of gene expression in anxiety disorders/traits, and providing a start point for mechanistic investigation and future drug development.

## Methods

### Genome-Wide Association Studies Summary Statistics

The GAD-2 anxiety GWAS summary statistics were from a recent large-scale GWAS of anxiety disorder (Levey et al., 2020) and were downloaded from dbGAP (https://dbgap.ncbi.nlm.nih.gov/, phs001672) (Levey et al., 2020). This GWAS was performed by the Million Veteran Program (MVP) which is a large biobank that includes information about genetic, environmental, and medical records (Gaziano et al., 2016). GAD-2 (Kroenke et al., 2010) was used to measure the degree of anxiety. European Americans (*N* = 175,163) and African Americans (*N* = 24,448) were included in the MVP. Considering that gene expression weights data were based on European populations, only GWAS summary statistics from European Americans were used in this study. Samples were genotyped with customized MVP Affymetrix genotyping array. Details about sample information, phenotyping, data processing, and statistical analyses were provided in the original paper (Levey et al., 2020).

### Gene Expression References Panels

Two sets of gene expression weights (SNP-gene expression associations) were used to perform the TWAS analysis. The first gene expression reference panel was from the PsychENCODE (Gandal et al., 2018). Briefly, gene expression and genotypes of 1,321 individuals were downloaded from the PsychENCODE website (http://resource.psychencode.org) and gene expression weights (SNP-gene expression relations) were generated by using the R script (FUSION.compute_weights.R) provided by the FUSION software. Genotype data from the PsychENCODE were used as the LD (linkage disequilibrium) reference panel (a file (PLINK format) (Purcell et al., 2007) which contains the genotype of a reference population that matches the GWAS sample ancestry. TWAS can use a personalized reference panel or the one provided by FUSION). The second expression panel was from GTEx (Battle et al., 2017) (V7) and only expression weights from brain tissues (a total of 13) were used. For TWAS using the GTEx dataset, the LD reference panel (from European ancestry) was downloaded from the FUSION website (https://data.broadinstitute.org/alkesgroup/FUSION/LDREF.tar.bz2) provided by Gusev et al. (2016) More detailed information about FUSION software, PsychENCODE, and GTEx were provided in the original papers (Gusev et al., 2016).

### Transcriptome-Wide Association Study

To identify genes whose genetically-regulated expression are associated with risk of anxiety, we performed TWAS analyses by integrating GWAS summary statistics of anxiety and gene expression weights from the PsychENCODE and GTEx by using FUSION software (http://gusevlab.org/projects/fusion/) (Gusev et al., 2016) (with the using of default settings). The basic process were as follows: Firstly, FUSION computed TWAS expression weights (i.e., SNP-gene expression correlations) using five linear models, including BLUP, BSLMM, LASSO, Elastic Net, and top SNPs from the reference expression panels (i.e., PsychENCODE and GTEx). When performing transcriptomic imputation, FUSION calculated an out-sample R^2^ using a fivefold cross-validation of each model to determine the best performing prediction model for a gene. Then the imputed gene expression was used to test the association with anxiety. Data from the PsychENCODE (http://resource.psychencode.org/#Derived) (Gandal et al., 2018) and Gusev *et al.* (https://data.broadinstitute.org/alkesgroup/FUSION/LDREF.tar.bz2) (Gusev et al., 2016) were used as LD reference panels to account for LD structure. Transcriptome-wide significant genes were corrected by the Bonferroni correction approach (*p* = 3.52 × 10^-06^, 0.05/14,223 (the number of genes in the weight files) for PsychENCODE. Variable *P* thresholds were applied to GTEx as the gene number included in different brain weight files were different. GTEx *P*_thresholds_ = 0.05/(the number of genes in each GTEx weight file)/13 (13 was the number of GTEx reference panels included in this study).

### Joint/Conditional Analysis

We performed Joint/Conditional analysis on transcriptome-wide significant (TWS) loci by utilizing the script FUSION.post_process.R implemented in FUSION (Gusev et al., 2016). The Joint/Conditional analysis aimed at exploring to what extent the GWAS signals remain significant after removing TWAS significant signals (i.e., test if the GWAS signals are still significant after removing the expression weights from TWAS). This analysis requires the top significant genes from TWAS analysis, LD reference panel, and GWAS summary statistics as input. Each SNP association from anxiety GWAS was conditioned on the joint model (one SNP at a time) and permutation test (100,000) was used to determine the *p*-value of Joint/Condition analysis results. The genes that passed the permutation test represent promising driven genes (i.e., these genes are unlikely to be co-localized due to chance). Our TWAS analysis pipeline is in agreement with the original FUSION paper. Default settings and parameters (recommended by FUSION software) were used in Joint/Conditional analysis.

### Tissue and Cell-Type Enrichment Analysis

To explore if the genetic associations identified by anxiety GAD-2 GWAS were enriched in specific tissues and cell types, we utilized MAGMA (de Leeuw et al., 2015) (implemented in FUMA (Watanabe et al., 2017)) (https://fuma.ctglab.nl/) to conduct tissue and cell-type-specific enrichment analysis (with the using of default parameters and settings). FUMA detected tissue-specific enrichment of genome-wide associations using expression data from 53 GTEx tissues (Battle et al., 2017). Briefly, FUMA first defined the differentially expressed genes (DEGs) for each tissue (by comparing the expression level of a gene in a specific tissue to all other tissues). The defined DEGs for each tissue contained genes with the greatest expression discrepancy in the specific tissue compared with other tissues. These DEGs were then used as input in enrichment analysis with MAGMA. Details, principles, and procedures have been described in the original paper (Watanabe et al., 2017) and the FUMA website.

Cell-type enrichment analysis was also performed by MAGMA (de Leeuw et al., 2015). The single-cell gene expression data of the mouse central nervous system was obtained from Zeisel et al. (2018). Data was processed following the instructions by Bryois et al. (2020). Gene expression data of 160,769 single cells were analyzed and genes that were not expressed were removed. We calculated gene expression specificity as described by Bryois et al. (2020), and the top 10% genes ranked by gene expression specificity of each cell was remained for MAGMA gene set analysis. Mouse gene ids were mapped to their corresponding human genes (one-to-one orthologous) based on MGI annotations (http://www.informatics.jax.org/homology.shtml). FDR (false discovery rate) was used for multiple correction.

### Pathway and Gene Ontology Enrichment Analysis

We utilized gene network v2.0 (https://genenetwork.nl/) (Deelen et al., 2019) to analyze the co-expression pattern and to investigate if TWS genes are enriched in specific pathways/GO terms. GeneNetwork contains a gene expression matrix from public RNA-seq datasets with 31,499 samples and 56,435 genes. Firstly, a PCA (principal component analysis) was performed on this gene expression matrix, and 1,588 principal components were selected. Secondly, for each PC, a t-test was performed for genes that belonged to a GO term/pathway and other genes. Thirdly, a Mann–Whitney U test was performed to determine the Z-score of the annotated genes and genes that were not in the network. Gene sets including REACTOME, GO, and KEGG pathways were used. For details on RNA-seq data processing, PCA, co-regulation analysis, and other statistical analysis, please refer to the original paper of network v2.0^39^

## Results

### Transcriptome-Wide Association Study Identified 26 Risk Genes for Anxiety

**FIGURE 1:**
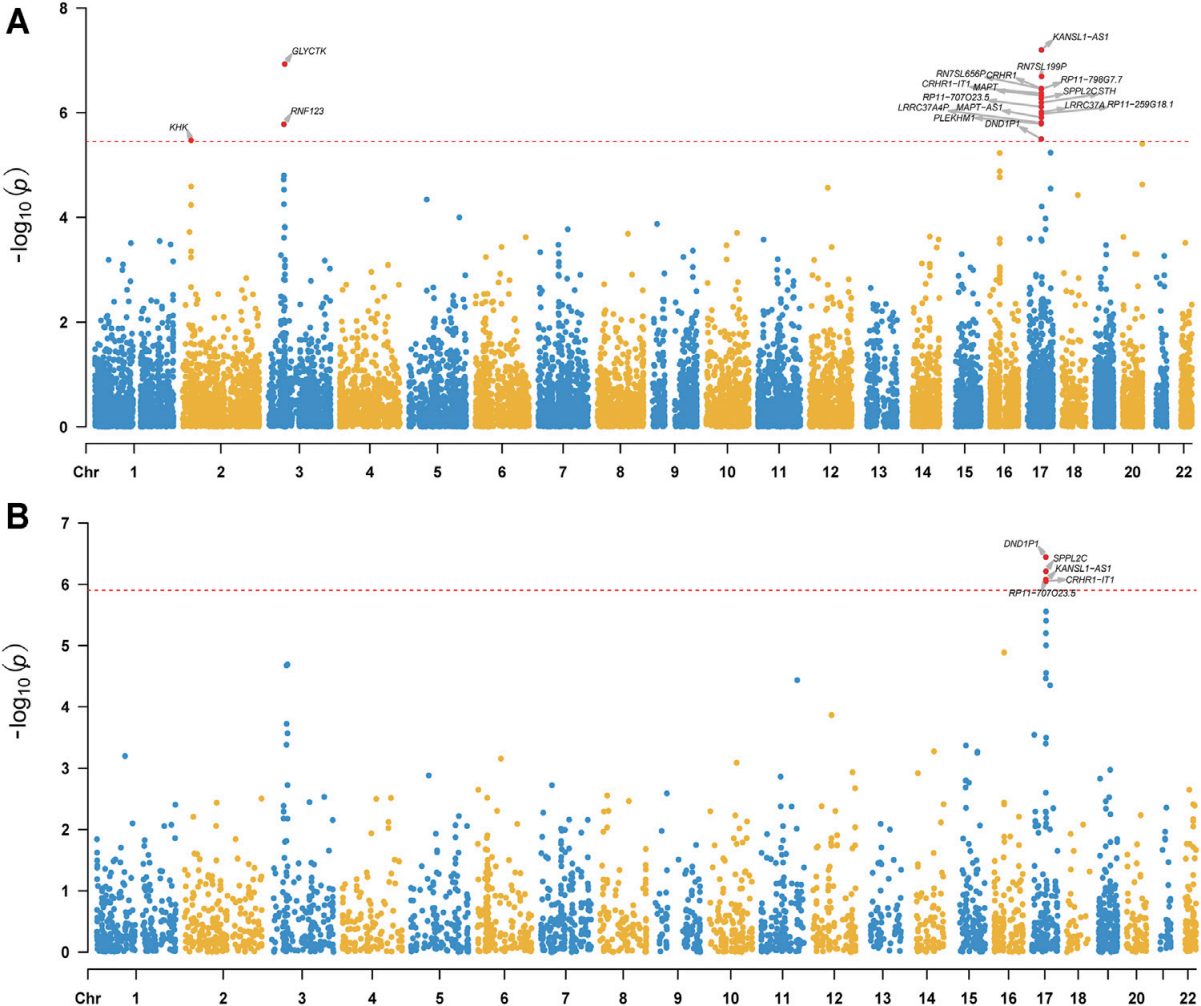
Manhattan plot of TWAS results of anxiety (gene expression reference was from the PsychENCODE **(A)** and GTEx Frontal Cortex BA9 **(B)**. The y-axis is the −log_10_(*P*) of TWAS (generated by FUSION) result. The red dash line indicates the Bonferroni-corrected significant level. Gene reached transcriptome-wide significance are shown.

We conducted TWAS by integrating GAD2-GWAS summary statistics of anxiety and two sets of brain gene expression reference panels (i.e., eQTL data) from PsychENCODE and GTEx. We identified 19 TWS genes (from four genomic loci, 2p23.3, 3p21.31, 3p21.1 and 17q21.31) when PsychENCODE brain eQTL data were used (Figure 1A; Table 1; Supplementary Table S1). Of note, genes from these four genomic loci did not show genome-wide associations with anxiety in the original GWAS (Levey et al., 2020).

**TABLE 1:** Genes that showed transcriptome-wide significant associations with anxiety in PsychENCODE or GTEx brain eQTL datasets.

Gene	Region	Lead SNP(*p* value)^a^	PsychENCODE				GTEx
			Z^b^	P	Z	P	Tissue^c^
*KANSL1-AS1* ^d^	17:44270942–44274089	rs142925250(5.95E−08)	5.41	6.28E−08	5.33	9.87E−08	Hypothalamus
*GLYCTK* ^e^	3:52321105–52329272	rs13094687(4.38E−06)	5.30	1.17E−07	4.64	3.45E−06	Anterior_cingulate_cortex_BA24
*RN7SL199P* ^e^	17: 44614900–44615180	rs142925250(5.95E−08)	5.20	2.02E−07	NA	NA	NA
*CRHR1* ^d^	17: 43699267–43913194	rs142925250(5.95E−08)	−5.10	3.44E−07	−4.91	9.11E−07	Putamen_basal_ganglia
*RN7SL656P* ^e^	17: 44397045–44397325	rs142925250(5.95E−08)	5.10	3.47E−07	NA	NA	NA
*RP11-798G7.7* ^e^	17: 43627147–43636104	rs142925250(5.95E−08)	5.09	3.52E−07	NA	NA	NA
*CRHR1-IT1* ^d^	17: 43697694–43725582	rs142925250(5.95E−08)	−5.06	4.27E−07	5.23	1.72E−07	Nucleus_accumbens_basal_ganglia
*MAPT* ^e^	17: 43971748–44105700	rs142925250(5.95E−08)	−5.04	4.72E−07	−4.61	4.05E−06	Cerebellum
*SPPL2C* ^d^	17: 43922256–43924438	rs142925250(5.95E−08)	−5.02	5.29E−07	5.32	1.05E−07	Cerebellar_Hemisphere
*STH* ^e^	17: 44076616–44077060	rs142925250(5.95E−08)	−4.98	6.40E−07	NA	NA	NA
*RP11-707O23.5* ^d^	17: 43678235–43679706	rs142925250(5.95E−08)	4.94	7.71E−07	4.94	7.65E−07	Spinal_cord_cervical
*LRRC37A* ^e^	17: 44370099–44415160	rs142925250(5.95E−08)	4.90	9.75E−07	4.18	2.89E−05	Cerebellum
*RP11-259G18.1* ^d^	17: 44344403–44346060	rs142925250(5.95E−08)	−4.88	1.05E−06	4.92	8.71E−07	Caudate_basal_ganglia
*MAPT-AS1* ^d^	17: 43921017–43972966	rs142925250(5.95E−08)	−4.85	1.23E−06	5.03	4.93E−07	Cerebellar_Hemisphere
*LRRC37A4P* ^d^	17: 43578685–43627701	rs142925250(5.95E−08)	−4.80	1.56E−06	−5.25	1.53E−07	Substantia_nigra
*PLEKHM1* ^d^	17: 43513266–43568115	rs142925250(5.95E−08)	−4.80	1.62E−06	−4.96	6.89E−07	Cerebellar_Hemisphere
*RNF123* ^e^	3: 49726932–49758962	rs111488606(4.08E−07)	4.80	1.66E−06	4.55	5.25E−06	Hypothalamus
*DND1P1* ^d^	17: 43663237–43664295	rs142925250(5.95E−08)	−4.66	3.16E−06	5.10	3.44E−07	Anterior_cingulate_cortex_BA24
*KHK* ^e^	2: 27309615–27323640	rs1057394(3.25E−06)	−4.65	3.35E−06	−3.56	3.66E−04	Putamen_basal_ganglia
*RP11-259G18.2* ^f^	17: 44320972–44322410	rs142925250(5.95E−08)	NA	NA	4.87	1.09E−06	Anterior_cingulate_cortex_BA24
*RP11-798G7.5* ^f^	17: 43580626–43612076	rs142925250(5.95E−08)	3.65	2.62E−04	−5.18	2.22E−07	Cerebellar_Hemisphere
*RP11-798G7.8* ^f^	17: 43608943–43611204	rs142925250(5.95E−08)	2.56	1.06E−02	5.08	3.77E−07	Cerebellar_Hemisphere
*RGS19* ^f^	20: 62704534–62711323	rs6062344(2.69E−08)	4.23	2.35E−05	5.65	1.61E−08	Cerebellum
*RP11-259G18.3* ^f^	17: 44336917–44337972	rs142925250(5.95E−08)	NA	NA	5.14	2.74E−07	Cerebellum
*AMT* ^f^	3: 49454211–49460186	rs111488606(4.08E−07)	−0.68	4.98E−01	−4.88	1.08E−06	Hypothalamus
*ARHGAP27* ^f^	17: 43471275–43511787	rs142925250(5.95E−08)	2.65	8.03E−03	4.99	6.18E−07	Nucleus_accumbens_basal_ganglia

a The GAD2-score GWAS *p* value of lead SNPs of five TWS regions (17q21.31, 2p23.3, 20q13.33, 3p21.31, 3p21.1).

b Z reflects the association strength between this gene and anxiety, a positive value indicates up-regulation of this gene is predicted to be associated with increased GAD-symptom intensity.

c GTEx brain tissues with the lowest *p* value were shown in the table.

d Genes that show both TWS (Transcriptome-wide significant) in PsychENCODE and GTEx dataset.

e Genes that only show TWS in PsychENCODE dataset.

f Genes that only show TWS in GTEx dataset.

Though the sample size of the PsychENCODE is large, most of the brain tissues used for eQTL analysis were from the dorsolateral prefrontal cortex. We thus further carried out TWAS using brain SNP-gene expression weights (SNP-gene expression relations) from 13 GTEx brain tissues (Battle et al., 2017). A total of 17 TWS genes spanning three genomic loci **(**3p21.31, 17q21.31, 20q13.33) were identified in this analysis (Figure 1B, Table 1, Supplementary Figure S1–S12, Supplementary Table S2). Intriguingly, genes from two genomic loci did not show genome-wide significant associations with anxiety in the original GWAS. We compared the results from these two analyses and identified 10 overlapping genes that showed transcriptome-wide significant associations with anxiety in both datasets (Table 1). These overlapping genes include *KANSL1-AS1*, *CRHR1, CRHR1-IT1, SPPL2C, RP11-707O23.5, RP11-259G18.1, MAPT-AS1, LRRC37A4P, PLEKHM1,* and *DND1P1*. These findings identified genes whose genetically regulated expression may confer risk of anxiety, suggesting that genetic variants may confer anxiety risk by regulating the expression of these genes.

### Conditional Analysis of Transcriptome-Wide Association Study Significant Loci

**FIGURE 2:**
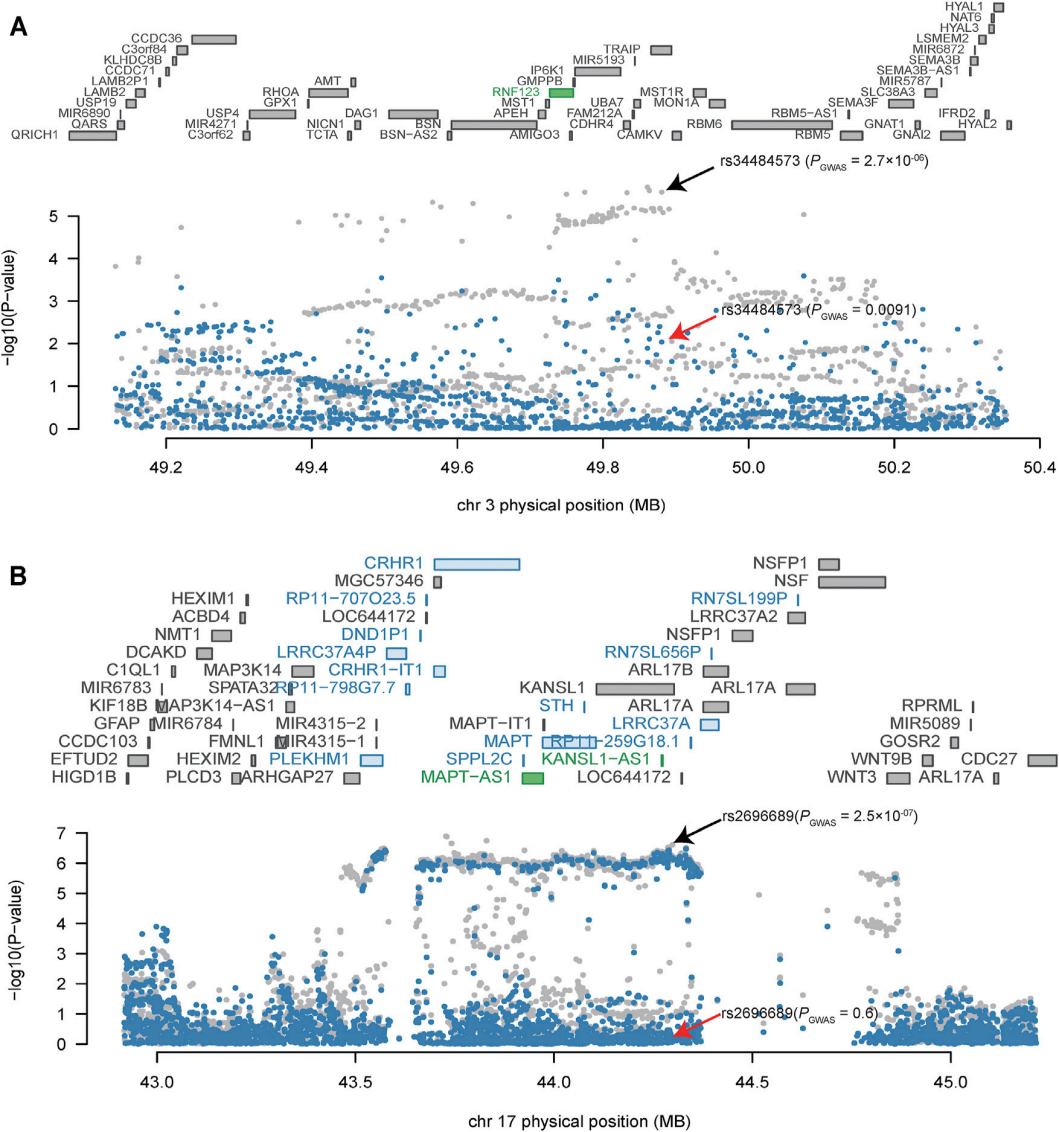
Conditional analysis of TWAS significant loci. **(A)** Conditional analysis of TWAS significant locus on 3p21.31. **(B)** Conditional analysis of TWAS significant locus on 17q21.31. The top panel of the Joint/conditional plot is all genes that located in the loci (usually gray), the genes with marginally TWAS association were marked in blue, genes that are jointly significant are in green. The bottom panel is the Manhattan plot of the original GWAS summary statistics data before (gray) and after (blue) conditioning on the green genes. The arrows indicate the association result of lead SNPs before (black arrow) and after (red arrow) conditional/joint analysis.

As several TWS genes were identified in each risk locus, we next sought to explore which gene drove the TWAS signal (i.e., to test if the transcriptome-wide association signal is conditionally independent or not). We therefore performed Joint/Conditional tests for TWAS significant regions on 3p21.31, and 17q21.31, two regions supported by TWAS of two different brain eQTL datasets (PsychENCODE and GTEx). We identified several independent transcriptome-wide significant genes from both of the brain eQTL datasets. For example, we found that *RNF123* explains most of the variance (0.725) at its locus in PsychENCODE dataset (rs34484573 lead SNP *P*_GWAS_ = 2.7 × 10^-06^, conditioned on *RNF123* lead SNP *P*_GWAS_ = 0.0091) (Figure 2A). In addition, two genes on 17q21.31, including *MAPT−AS1*and *KANSL1−AS1* (Figure 2B), are also jointly significant in PsychENCODE dataset. These two genes jointly explain most (0.894) a large proportion of the variance at this locus (rs2696689 lead SNP *P*_GWAS_ = 2.5 × 10^-07^, conditioned on *MAPT-AS1* and *KANSL1−AS1* lead SNP *P*_GWAS_ = 0.60). In addition, the 17q21.31 region also showed TWS in GTEx frontal cortex (BA9) region (Figure 1B), the TWS gene *DND1P1* explained the most of the variance (0.951) in this loci (rs17631676 lead SNP *P*_GWAS_ = 7.2 × 10^-07^, conditioned on *DND1P1* lead SNP *P*_GWAS_ = 0.27) (Supplementary Figure S13).

### Anxiety Associations Were Enriched in Brain Tissues and Neurons

**FIGURE 3:**
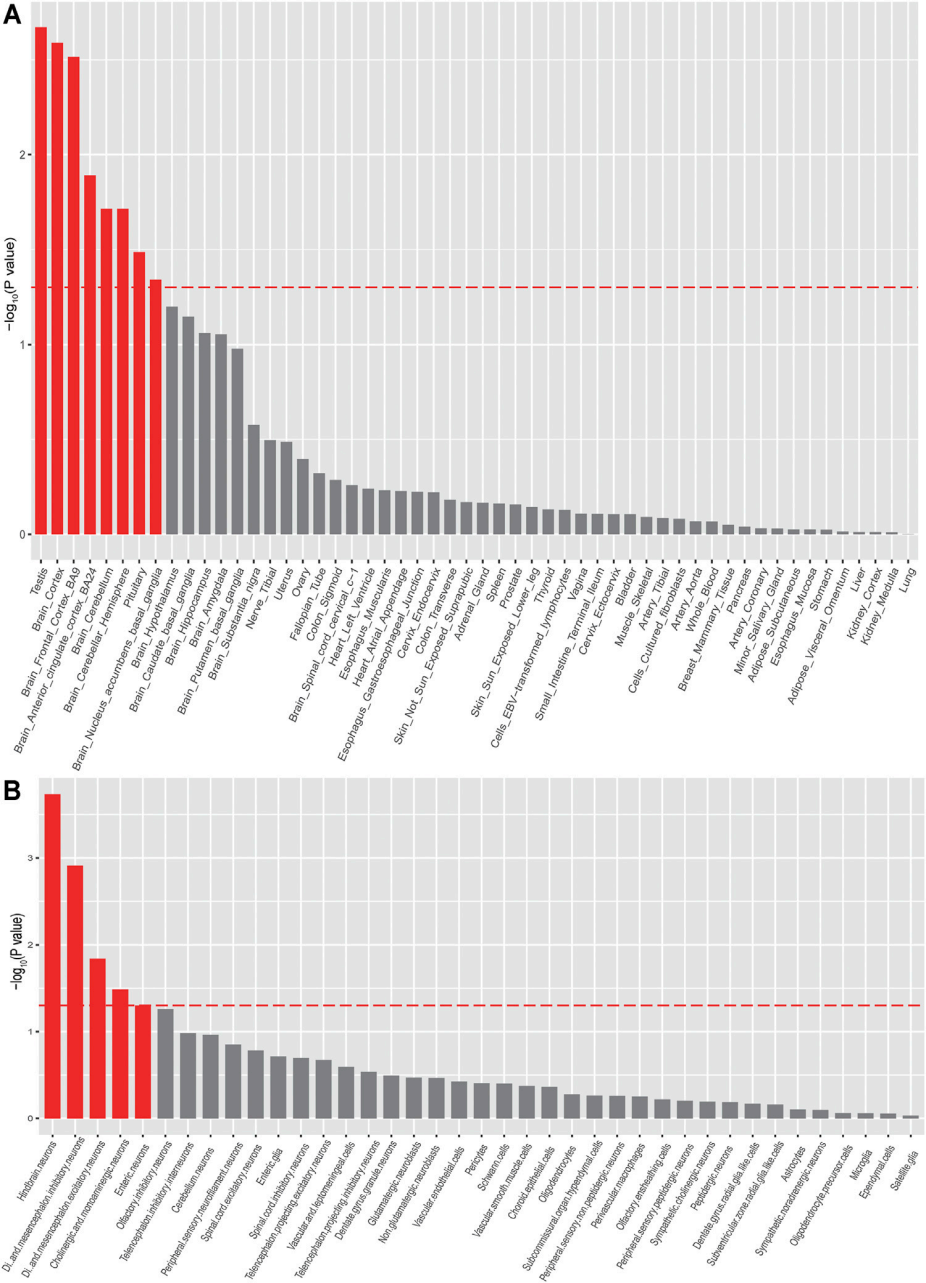
Tissue and cell-type enrichment analysis of anxiety disorder summary statistics (MAGMA). Tissues and cell types that showed significant enrichment (*p* < 0.05) were marked in red.

To explore if the genome-wide associations of anxiety were enriched in specific tissues, we performed tissue-specific enrichment analysis using MAGMA (de Leeuw et al., 2015) (implemented in FUMA (Watanabe et al., 2017) (Figure 3, Supplementary Table S3). The MVP GAD-2 score anxiety GWAS summary statistics were used as input for MAGMA. Overall, we found significant enrichment of anxiety associations in brain tissues (Figure 3, Supplementary Table S3), including the cortex (*p* = 0.0026), frontal cortex (*p* = 0.0031), anterior cingulate cortex (*p* = 0.013), etc. Interestingly, we noticed that anxiety associations were also enriched in the testis (*p* = 0.0021). We then investigated the enrichments of anxiety associations in different brain cell types and found significant enrichments in neurons from the hindbrain (*p* = 0.00018), inhibitory (*p* = 0.0012), and excitatory (*p* = 0.014) neurons, as well as cholinergic and monoaminergic neurons (*p* = 0.026). These data prioritized the possible tissues and cell types that were affected by anxiety risk genes (Figure 3, Supplementary Table S4).

### Potential Biological Relevance of Transcriptome-Wide Association Study Significant Genes

To explore the potential biological implications of TWAS findings, we utilized the gene network (V2) (Deelen et al., 2019) (https://genenetwork.nl/gene-list/) to carry out gene network analysis. The genes were clustered based on the co-expression relationship based on public RNA-seq data (*n* = 31,499). Our gene network analysis showed that the 10 TWS genes (in both PsychENCODE and GTEx datasets) (Table 1) formed two clusters (or networks) based on their co-expression pattern (Supplementary Figure S14). The first cluster includes *CRHR1, LRRC37A4P, KANSL1-AS1, SPPL2C, DND1P1, MAPT-AS1.* And the other cluster contains *CRHR1-IT1, RP11-259G18.1, PLEKHM1,* and *RP11-707O23.5*. These results suggest that anxiety risk genes tend to form network to exert their biological effects.

We next performed pathway and GO Enrichment analysis (based on REACTOME, GO, KEGG) to test if the TWS genes were enriched in specific biological pathways (Table 2). REACTOME enrichment analysis showed that the TWS genes are enriched in myogenesis processes, including CDO in myogenesis (*p* = 2.8 × 10^-4^) and myogenesis (*p* = 2.8 × 10^-4^). Of note, GO analysis showed that the TWS genes were significantly enriched in the GO biological process Wnt signaling pathway, calcium modulating pathway (*p* = 1.9 × 10^-03^), indicating that risk variants may confer risk of anxiety by affecting these biological processes.

**TABLE 2:** Pathway and GO enrichment analysis result of TWAS significant genes (items with *p* < 0.01 were shown).

Pathway/GO	*p*-value	FDR	Database
CDO in myogenesis	0.00028	0.99	REACTOME
Myogenesis	0.00028	0.99	REACTOME
Interleukin-17 signaling	0.01	0.99	REACTOME
Ca^2+^ pathway	0.01	0.99	REACTOME
Phagocytic vesicle	0.01	0.98	GO cellular component
ligand-gated ion channel activity	0.0088	0.96	GO molecular function
mRNA binding	0.0004	0.16	GO molecular function
Structural constituent of eye lens	0.0029	0.57	GO molecular function
Wnt signaling pathway, calcium modulating pathway	0.0019	1.00	GO biological process
stress-activated protein kinase signaling cascade	0.0028	1.00	GO biological process
blood coagulation, intrinsic pathway	0.0034	1.00	GO biological process
regulation of membrane potential	0.0037	1.00	GO biological process
negative regulation of cell death	0.0049	1.00	GO biological process

## Discussion

In this study, we reported the first TWAS of anxiety to identify genes whose genetically regulated expression may confer risk of anxiety. Our study identified 26 TWS genes, of which 25 were not nominated in the original GWAS, indicating that TWAS is a powerful approach to nominate novel risk genes that are not implicated by GWAS. And 10 TWS genes were identified in both PsychENCODE and GTEx datasets. Among the 10 overlapping genes, we found that *LRRC37A4P* is widely expressed in several neuronal cell types. None of these genes showed cell-type specific expression (Polioudakis et al., 2019) (Supplementary Figure S15–S20). Furthermore, we also found that genes located in 3p21.31 and 17q21.31 are significantly associated with anxiety. We noticed that these two loci also showed significant associations with other psychiatric disorders. For example, *MAPT* gene (located in 17q21.31) is a well-known candidate risk gene for Parkinson’s disease (Zabetian et al., 2007; Simón-Sánchez et al., 2009; Edwards et al., 2010). In addition, the 3p21.31 and 17q21.31 are also significantly associated with Post-traumatic stress disorder (PTSD) in a recent GWAS study by the MVP (Gelernter et al., 2019). Intriguingly, a recent TWAS on PTSD also indicated that genes located in these two regions are related to PTSD (Girgenti et al., 2021). Notably, *RNF123* was one of the top genes that were significantly associated with PTSD at the transcriptome-wide significance level (Girgenti et al., 2021). The shared TWAS findings may be due to the high genetic correlation (approximately 50–60%) between PTSD and anxiety (Stein et al., 2021). In addition, *RNF123* was also reported to be dysregulated in depression and may serve as a clinical biomarker for depression (Glahn et al., 2012; Teyssier et al., 2013). These results collectively indicate that *RNF123* may play an important role in stress-related disorders, including anxiety, PTSD, and depression. Though anxiety disorder, PTSD, and depression are diagnosed as different mental disorders (as they represent potentially fundamentally different dimensional endophenotypes (Insel and Cuthbert, 2009)), the genetic risk factors that these diseases shared may represent general genetic risk factors of these mental diseases (Smoller, 2016). Taken together, these lines of evidence indicate that these two genomic regions may harbor authentic risk genes for anxiety and genetic variants likely confer risk of anxiety by modulating the expression of genes located in 3p21.31 and 17q21.31.

*RNF123* (Ring Finger Protein 123) encodes E3 ubiquitin-protein ligase which is a subunit of the Kip1 ubiquitination-promoting complex (KPC). Previous studies revealed the important function of *RNF123*, including regulation of cell cycle process (Kamura et al., 2004) and innate antiviral signaling (Wang et al., 2016). Besides, several studies also showed that *RNF123* may have a role in cancer, including aggressive glioblastoma and melanoma (Iida et al., 2017; Wang et al., 2020). However, the function of *RNF123* in the human central nervous system and the corresponding mechanisms about how it confers the risk of mental disorders remain to be investigated.

Two major reasons might account for the observations that some of the loci were significant in original GWAS but not in TWAS. Firstly, TWAS was designed to detect the gene-trait association by performing transcriptome imputation of GWAS data with a reference trained gene expression predictive model (constructed from a dataset with both genotype and gene expression) (Gusev et al., 2016; Wainberg et al., 2019). If the genetic variations in the GWS loci are not functionally as cis-regulation variations (which affect gene expression), TWAS is not able to nominate candidate genes in these loci. Secondly, the power of the TWAS may be limited by the sample size of the training set we used, e.g., the psychENCODE dataset we used in this study can pinpoint more TWS genes than a single GTEx panel. We also identified loci that were not significant in original GWAS but significant in TWAS. In TWAS analysis, we can identify TWS loci that are not nominated by GWS, which is the power and advantages of TWAS (Gusev et al., 2016; Liao et al., 2019). For TWS loci not reported by GWS, a possible reason is that the statistic power is not enough in the current GWAS. For example, the leading GWAS association signal in TWS locus (17q21.31) is rs142925250 (5.95E-08, Table 1), which is close to GWS level (5.00E-08). With the increase of sample size, rs142925250 may reach the genome-wide significance level. Finally, we also identified loci that reached GWS and TWS simultaneously. The GWAS result is not easy to interpret because of complex LD (linkage disequilibrium) (several linked genes may show associations with a GWAS trait). TWAS leverages a reference expression panel (samples with gene expression and genotype data simultaneously) to perform transcriptome imputation on a target GWAS dataset, and the predicted gene expression of the GWAS dataset was used to identify the gene-trait relationship. So TWAS can help to nominate gene-trait relationships by integrating eQTL data and GWAS data, which may help to explain the functions of the GWS loci (Wainberg et al., 2019).

As the predicted expression change of the TWS genes may contribute to disease risk, we also explored the expression of TWS genes in individuals with anxiety disorder and controls, using the published differential gene expression study by Wingo *et al.* (Wingo and Gibson, 2015) Gene expression in the blood of 157 anxiety disorder cases and 179 controls (GSE61672) were measured by Wingo *et al* (Wingo and Gibson, 2015). We performed differential expression analysis using NCBI GEO2R (Barrett et al., 2013), and found no overlap between the differentially expressed genes (DEGs) and our TWAS findings. We hypothesized that this might be due to the tissue difference between DEG analysis (blood) and TWAS (brain).

We performed MAGMA analysis for two purposes. First, the original GAD-2 GWAS papers did not perform MAGMA gene-set enrichment analysis to interpret the GWAS results (Levey et al., 2020). We therefore performed gene-set enrichment analysis by MAGMA (de Leeuw et al., 2015) to explore if genome-wide associations were enriched in specific tissues or cell types (using expression data from GTEx and single-cell RNA sequencing dataset). Second, we aimed at finding out the most-relevant tissues for TWAS analysis when using GTEx reference panels. Our MAGMA tissue enrichment analysis indicated that the brain tissues in GTEx were suitable for performing TWAS. As expected, we found that the anxiety associations were enriched in brain tissues and neurons. Of note, significant enrichment in the testis was also observed. A possible reason for this observation is that only limited genome-wide associations were reported by the original GWAS. Though the sample size of the MVP is relatively large (*N* = 175,163), we noticed that only five genome-wide significant (GWS) loci have been identified in the original GWAS of anxiety. The number of GWS risk loci for anxiety is quite small compared with other psychiatric disorders such as schizophrenia and depression. In addition to sample size, we found that the heritability were different among anxiety disorders (20–60%) (Polderman et al., 2015; Craske et al., 2017), SCZ (approximately 80%) (Sullivan et al., 2003), and depression (30–40%) (Sullivan et al., 2000). The heritability estimated from the GWAS is lower than the true heritability as GWAS can only capture the common variations (Manolio et al., 2009). The heritability of anxiety traits estimated from GAD-2 based GWAS is about 5.58% (*n* = 175,163) (Levey et al., 2020), the heritability of schizophrenia explained by GWAS is approximately 23–24% (*n* = 135,236) (Lam et al., 2019), and the heritability of depression estimated from GWAS is about 8.7% (*n* = 480,359) (Wray et al., 2018). These results suggest that the polygenetic nature varies among these diseases. Second, gene expression patterns are highly similar between human brain and testis (Guo et al., 2005), which may result in the observation of significant enrichment of anxiety associations in testis. Finally, it is possible that the anxiety risk genes also play a role in testis. More work is needed to explain this interesting observation.

Our study highlighted the importance of TWAS (which integrates gene expression data with GWAS summary) in nominating risk genes for anxiety. However, genetics can only explain a small proportion of anxiety disorders/traits, which indicates that environmental factors are also involved in anxiety disorders/traits. Among the various environmental factors, psychosocial stress plays a vital role in anxiety (Bartlett et al., 2017). For example, early life adversity (Nemeroff CB, 2004; Maccari et al., 2014; Wiegand et al., 2021) is an important environmental risk factor for social anxiety disorder. The stress environmental factors like early life adversity would trigger the DNA methylation change (Provençal and Binder, 2015), and epigenome-wide association study (EWAS) have reported many genes that showed epigenetic changes in anxiety disorders, including *CFAP46* (Ziegler et al., 2019)*, SLC43A2,* and *TNXB* (Wiegand et al., 2021)*.* Cognitive-behavioral therapy (CBT) (Barlow et al., 2000) is an useful psychosocial intervention for anxiety disorders and previous study has reported that the methylation level of *IL1R1* was changed in subjects of panic disorder (one of the anxiety disorders) treated with CBT (Ziegler et al., 2019). These findings indicate that epigenetic regulation also plays an import role in anxiety disorders/traits.

Our study has several limitations. Firstly, we only included the GAD-2 score based GWAS summary statistics from the MVP project in our current study. We compared the phenotyping methods of this GWAS and other published anxiety GWASs (Otowa et al., 2009; Erhardt et al., 2011; Otowa et al., 2012; Walter et al., 2013; Davies et al., 2015; Otowa et al., 2016; Dunn et al., 2017; Stein et al., 2017; Meier et al., 2019; Levey et al., 2020; Purves et al., 2020), and found that the phenotyping methods were not identical across studies. We believed that it would be best to perform an independent replication study based on the same phenotyping method. Secondly, though several TWS genes are identified, more efforts are needed to uncover the roles of these identified risk genes in anxiety etiology. Thirdly, although TWAS could identify candidate risk genes for anxiety, the causal variants in the GWAS/TWAS significant loci could not be pinpointed by TWAS. More efforts are needed to reveal the genetic mechanisms and pathogenesis of the genes in the GWAS/TWAS significant loci in anxiety.

In summary, our study uncovered the gene-trait relationships of anxiety traits for the first time. We identified 26 TWS genes for anxiety. Our results provide novel insight into anxiety disease etiology and facilitate further mechanism studies and future drug development.

## Data Availability

The original contributions presented in the study are included in the article/Supplementary Material, further inquiries can be directed to the corresponding authors.
