# Supplementary material for: Transcriptome-Wide Association Study Provides Insights Into the Genetic Component of Gene Expression in Anxiety
Source: Front Genet. 2021 Sep 28;12:740134. doi: 10.3389/fgene.2021.740134 (PMC8505959; doi:10.3389/fgene.2021.740134)
Supplement: Supplementary file 1 [file DataSheet1.doc]

**Supplementary material for**

**Transcriptome-wide association study provides insights into the genetic component of gene expression in anxiety**

Xi Su^1,2^, Wenqiang Li^1,2^, Luxian Lv^1,2^, Xiaoyan Li^3^, Jinfeng Yang^3,4^, Xiong-Jian Luo^3,4,5,6,*^, Jiewei Liu^3*^

^1^Henan Mental Hospital, The Second Affiliated Hospital of Xinxiang Medical University, Xinxiang, Henan 453002, China

^2^Henan Key Lab of Biological Psychiatry, International Joint Research  Laboratory  for Psychiatry and Neuroscience  of  Henan, Xinxiang Medical University, Xinxiang, Henan 453002, China

^3^Key Laboratory of Animal Models and Human Disease Mechanisms of the Chinese Academy of Sciences & Yunnan Province, Kunming Institute of Zoology, Chinese Academy of Sciences, Kunming, Yunnan 650204, China

^4^Kunming College of Life Science, University of Chinese Academy of Sciences, Kunming, Yunnan 650204, China

^5^Center for Excellence in Animal Evolution and Genetics, Chinese Academy of Sciences, Kunming 650204, China

^6^KIZ-CUHK Joint Laboratory of Bioresources and Molecular Research in Common Diseases, Kunming Institute of Zoology, Chinese Academy of Sciences, Kunming, Yunnan 650204, China

*To whom correspondence should be addressed: Key Laboratory of Animal Models and Human Disease Mechanisms, Kunming Institute of Zoology, Chinese Academy of Sciences, Kunming, Yunnan 650204, China; Tel: +86-871-68125413, Fax: +86-871-68125413, E-mail: [luoxiongjian@mail.kiz.ac.cn](mailto:luoxiongjian@mail.kiz.ac.cn) (XJL), [liujiewei@mail.kiz.ac.cn](mailto:liujiewei@mail.kiz.ac.cn) (JWL).


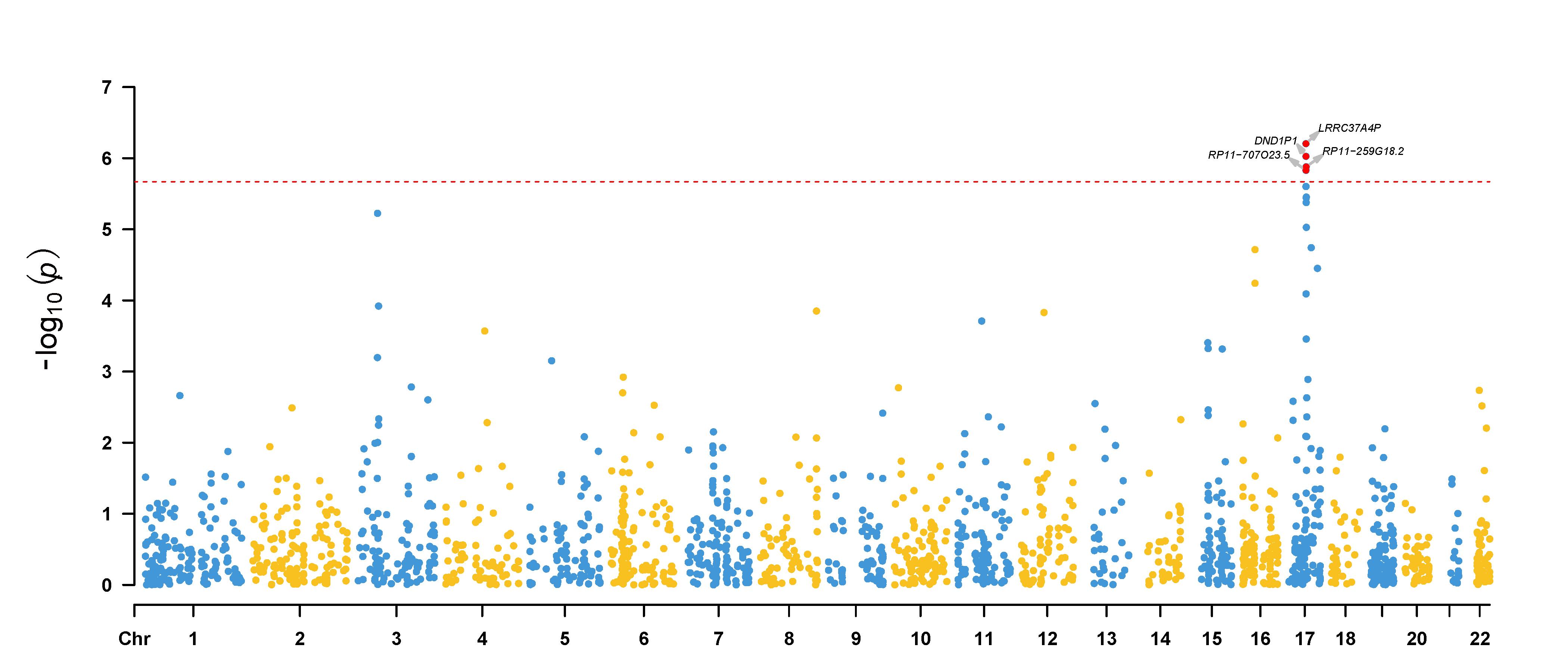


**Figure S1. Manhattan plot of TWAS results of anxiety (gene expression reference was from the GTEx Amygdala)**. The red dash line indicates the Bonferroni-corrected significant level.


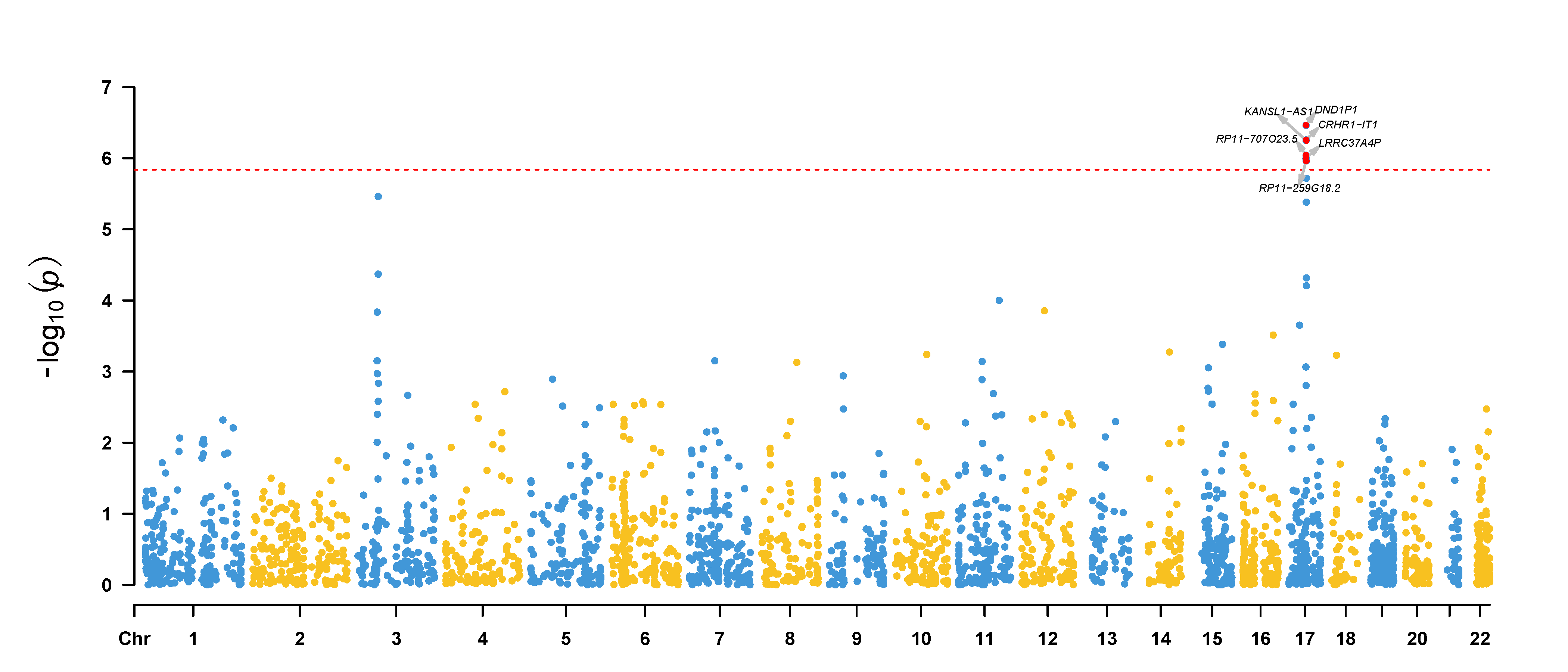


**Figure S2. Manhattan plot of TWAS results of anxiety (gene expression reference was from the GTEx Anterior cingulate cortex BA24)**. The red dash line indicates the Bonferroni-corrected significant level.


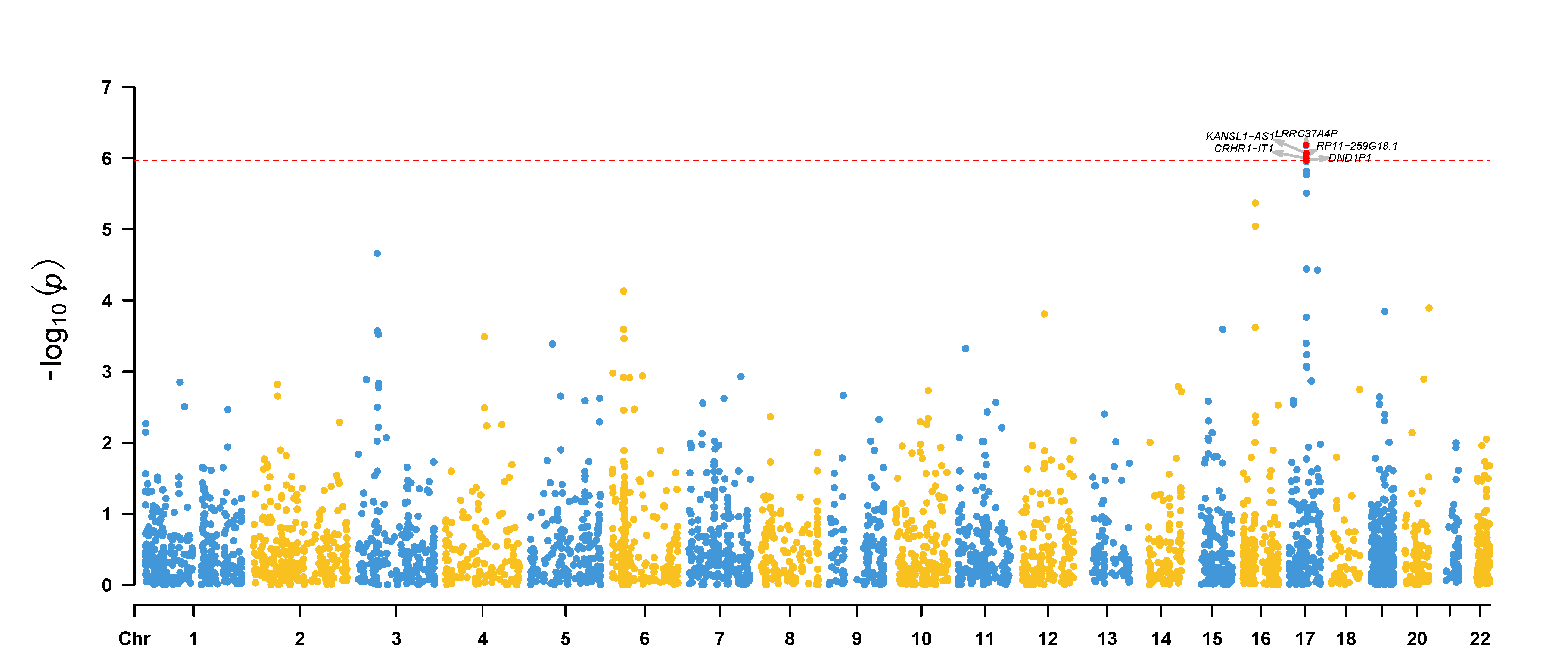


**Figure S3. Manhattan plot of TWAS results of anxiety (gene expression reference was from the GTEx Caudate_basal_ganglia)**. The red dash line indicates the Bonferroni-corrected significant level.


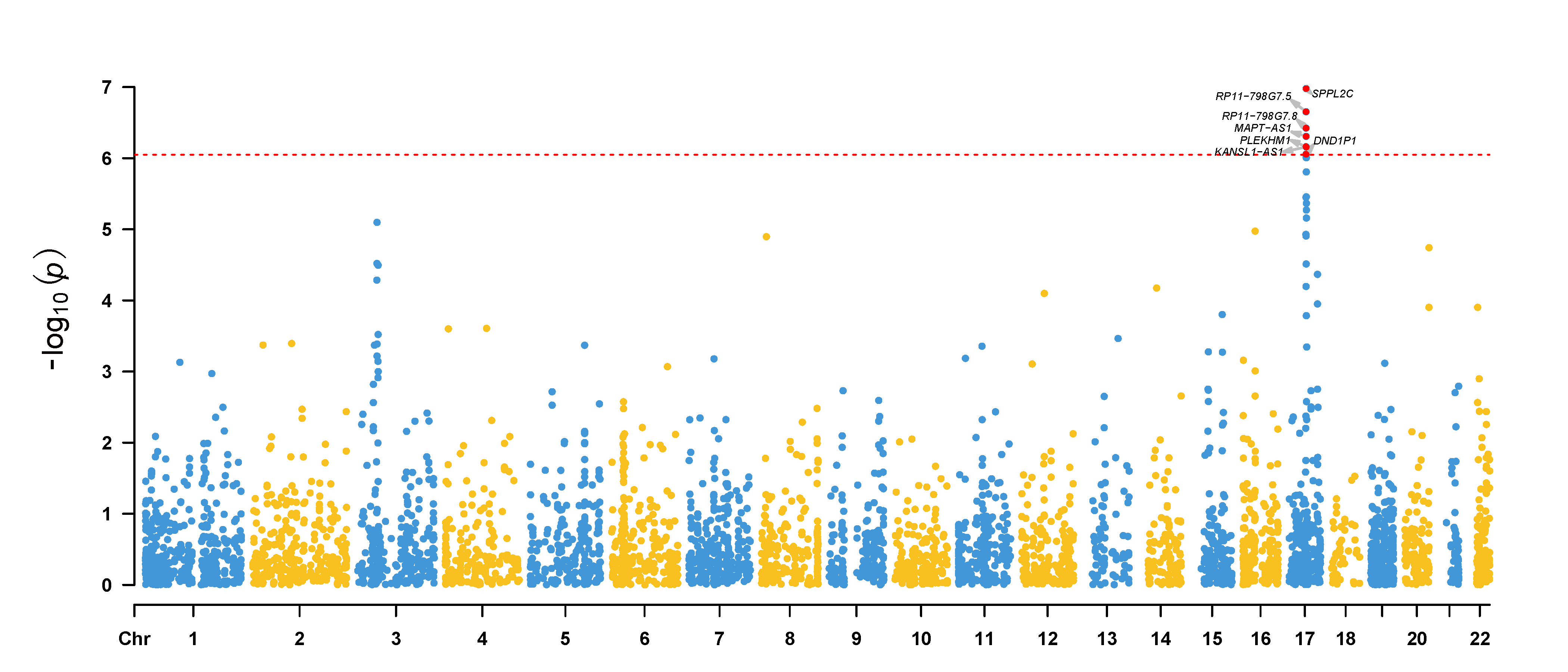


**Figure S4. Manhattan plot of TWAS results of anxiety (gene expression reference was from the GTEx**

**Cerebellar Hemisphere)**. The red dash line indicates the Bonferroni-corrected significant level.


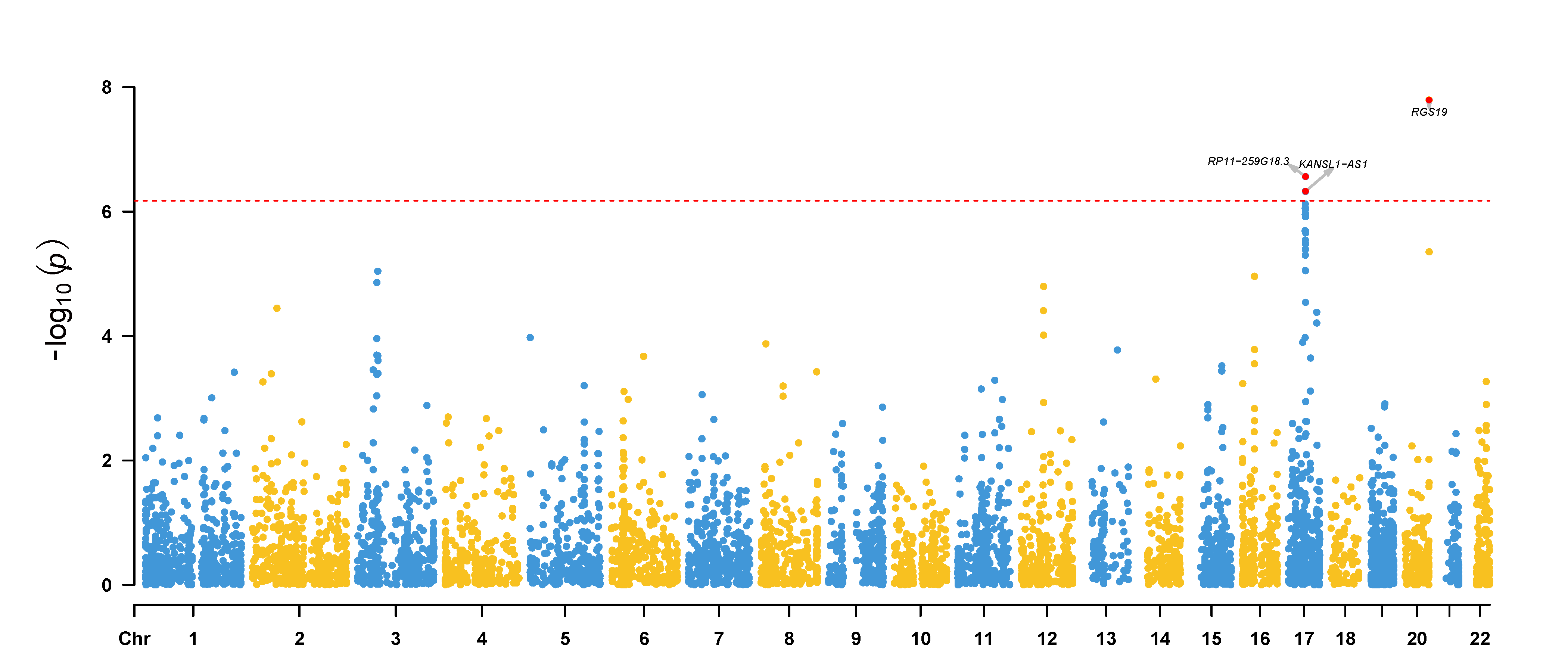


**Figure S5. Manhattan plot of TWAS results of anxiety (gene expression reference was from the GTEx Cerebellum).** The red dash line indicates the Bonferroni-corrected significant level.


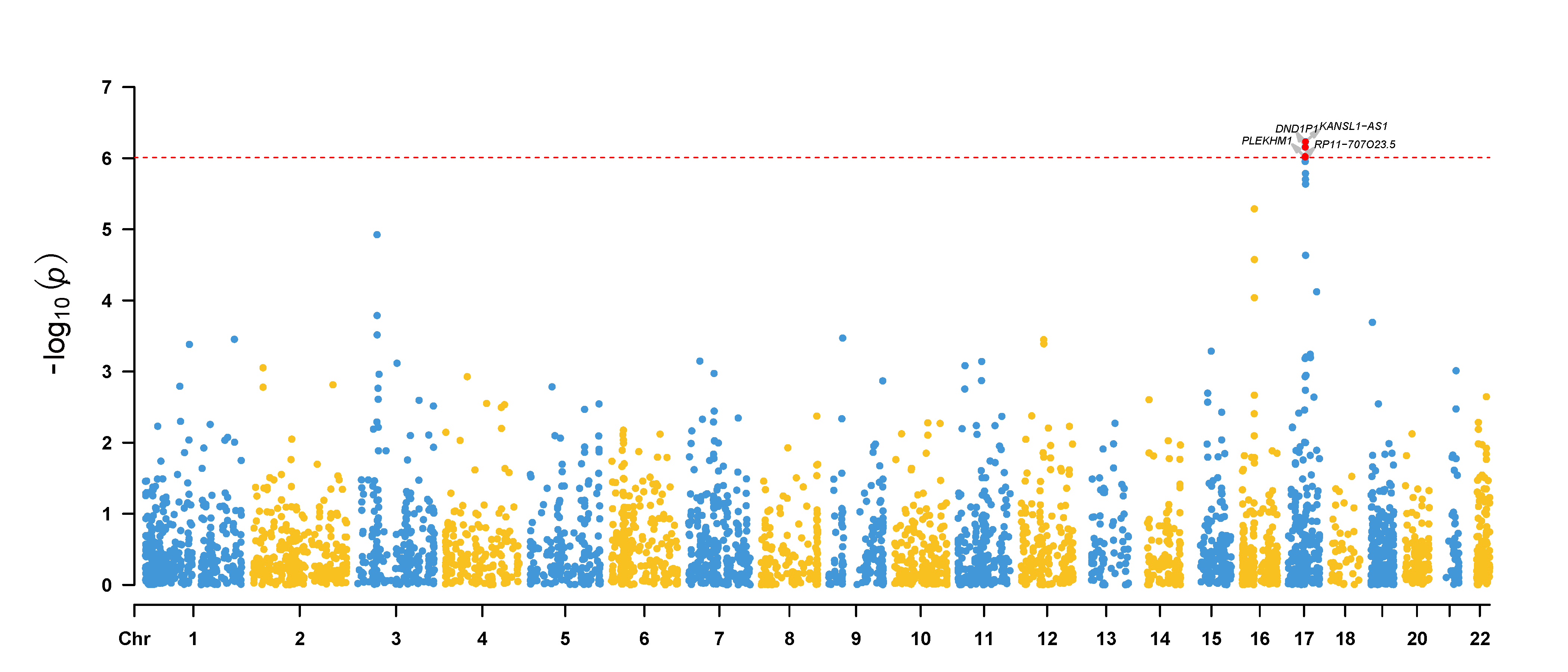


**Figure S6. Manhattan plot of TWAS results of anxiety (gene expression reference was from the GTEx Cortex).** The red dash line indicates the Bonferroni-corrected significant level.


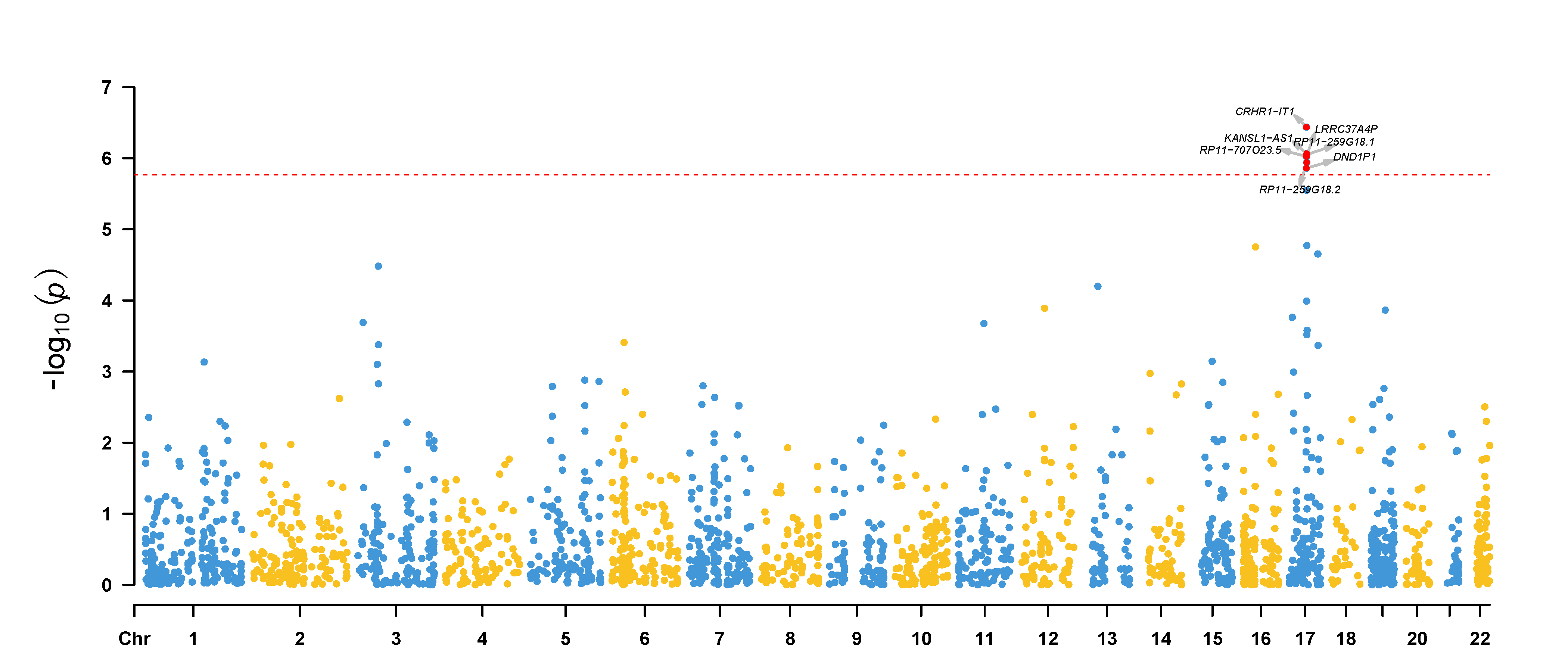


**Figure S7. Manhattan plot of TWAS results of anxiety (gene expression reference was from the GTEx Hippocampus).** The red dash line indicates the Bonferroni-corrected significant level.


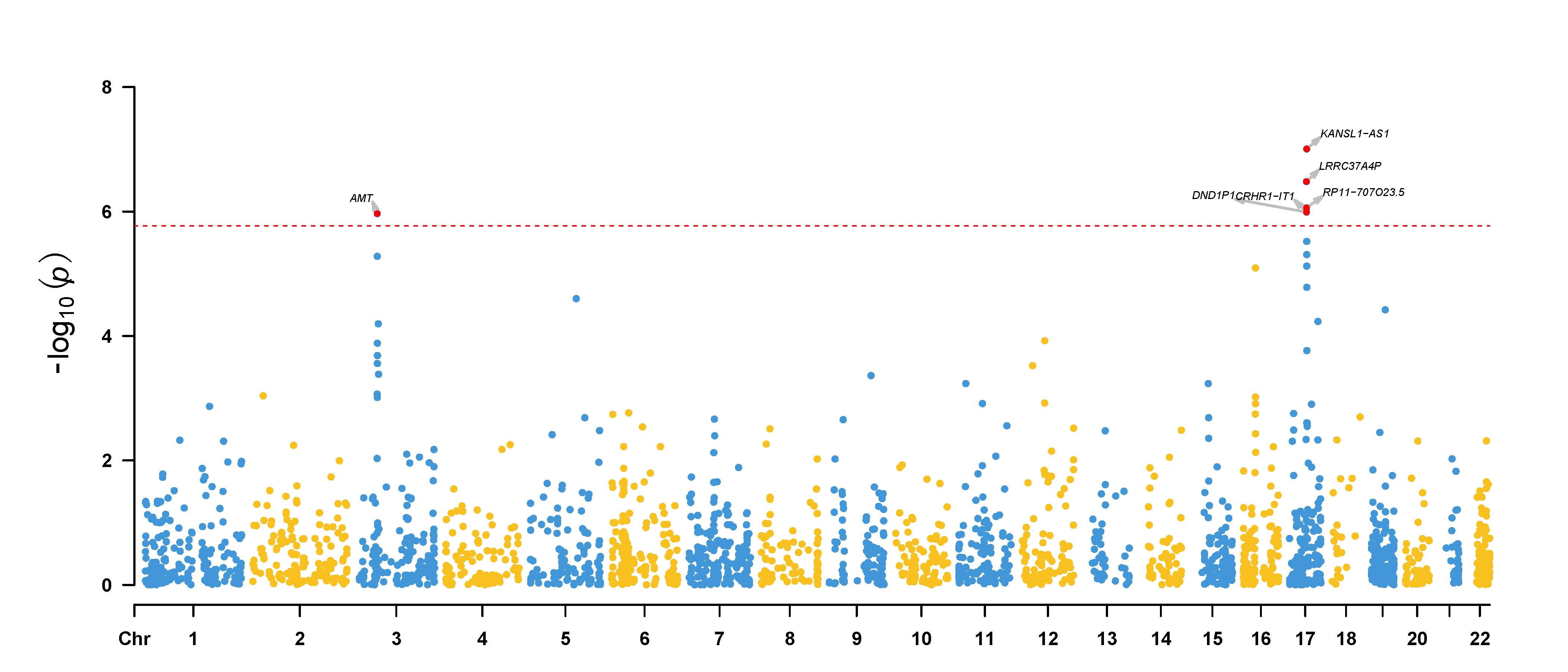


**Figure S8. Manhattan plot of TWAS results of anxiety (gene expression reference was from the GTEx Hypothalamus).** The red dash line indicates the Bonferroni-corrected significant level.


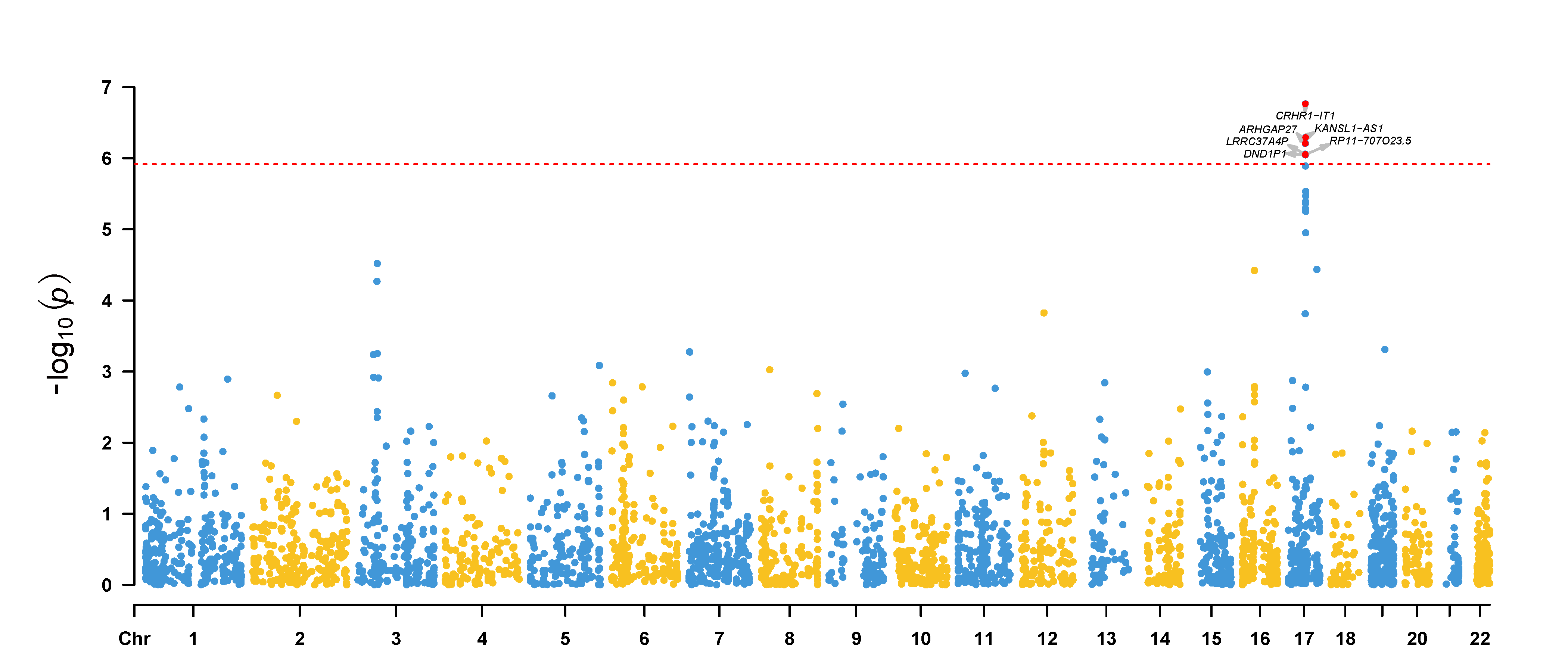


**Figure S9. Manhattan plot of TWAS results of anxiety (gene expression reference was from the GTEx Nucleus accumbens basal ganglia).** The red dash line indicates the Bonferroni-corrected significant level.


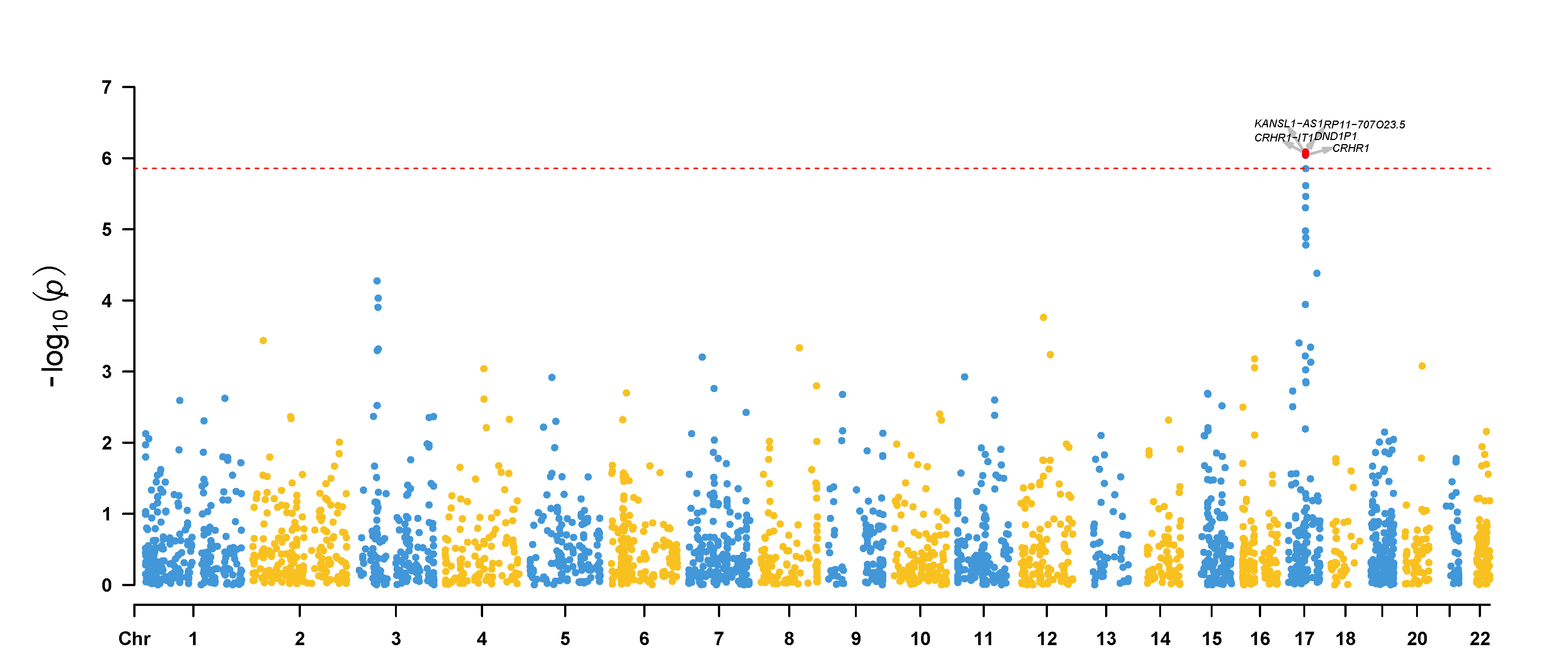


**Figure S10. Manhattan plot of TWAS results of anxiety (gene expression reference was from the GTEx Putamen).** The red dash line indicates the Bonferroni-corrected significant level.


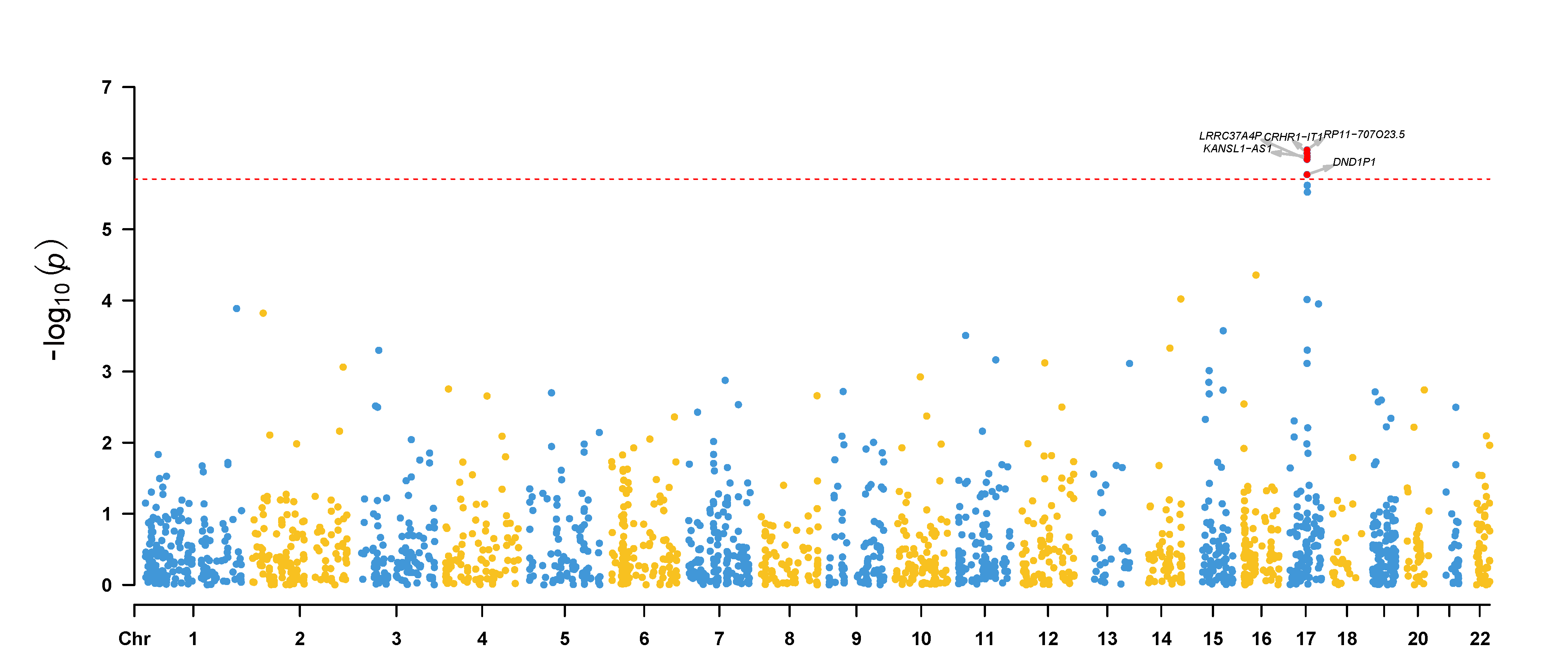


**Figure S11. Manhattan plot of TWAS results of anxiety (gene expression reference was from the GTEx Spinal cord cervical).** The red dash line indicates the Bonferroni-corrected significant level.


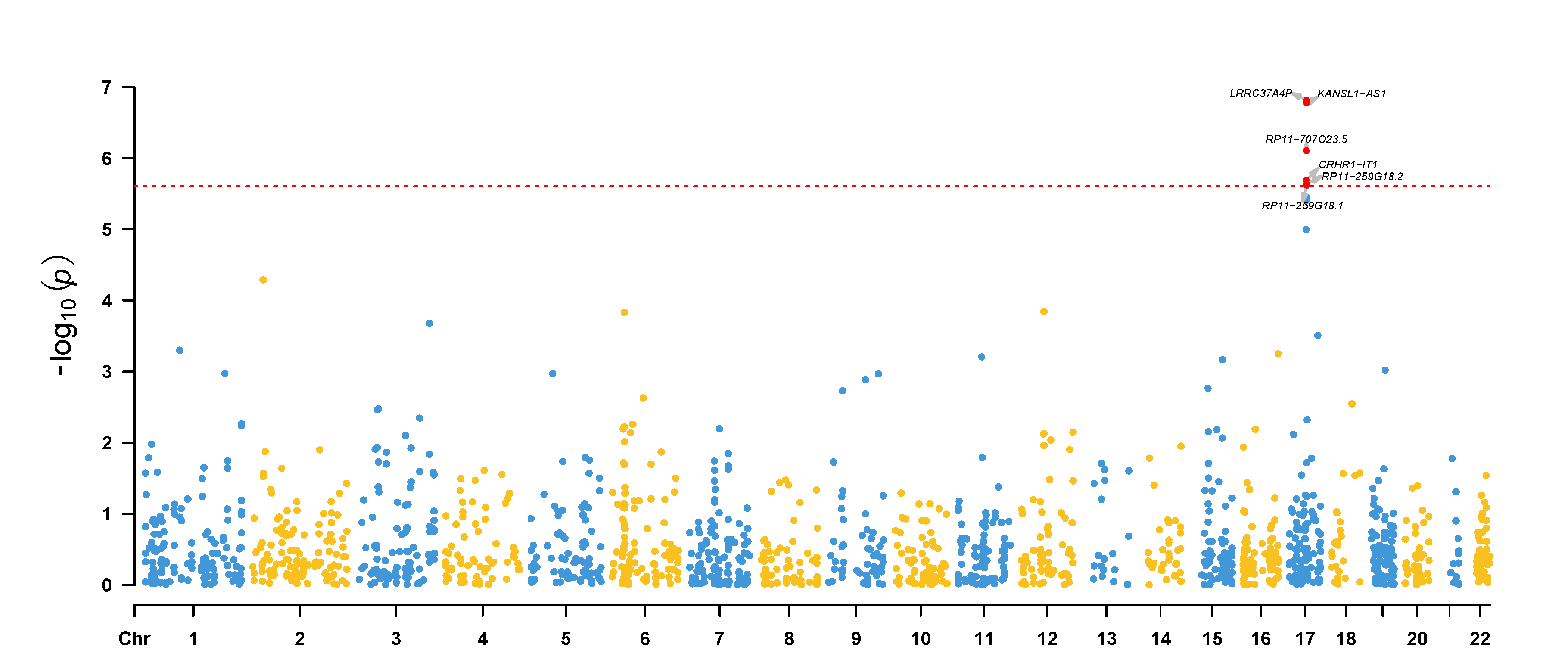


**Figure S12. Manhattan plot of TWAS results of anxiety (gene expression reference was from the GTEx Substantia nigra).** The red dash line indicates the Bonferroni-corrected significant level.


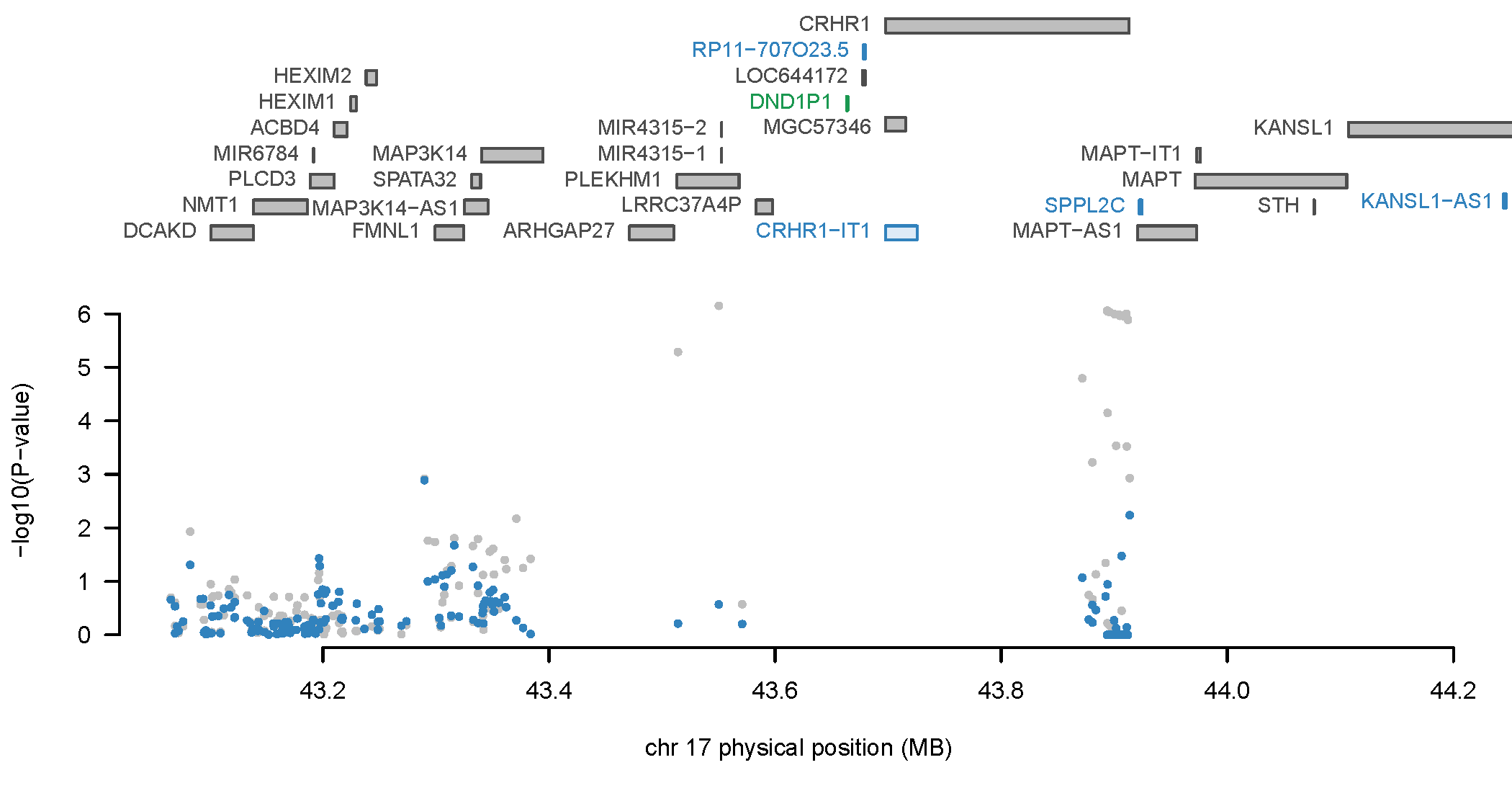


**Figure S13. Conditional analysis of TWAS significant locus on 17q21.31 (GTEx Frontal Cortex (BA9) panel).** The top panel of the Joint/conditional plot is all genes that located in the loci (usually gray), the genes with marginally TWAS association were marked in blue, genes that are jointly significant are in green. The bottom panel are Manhattan plot of the original GWAS summary statistics data before (gray) and after (blue) conditioning on the green genes.


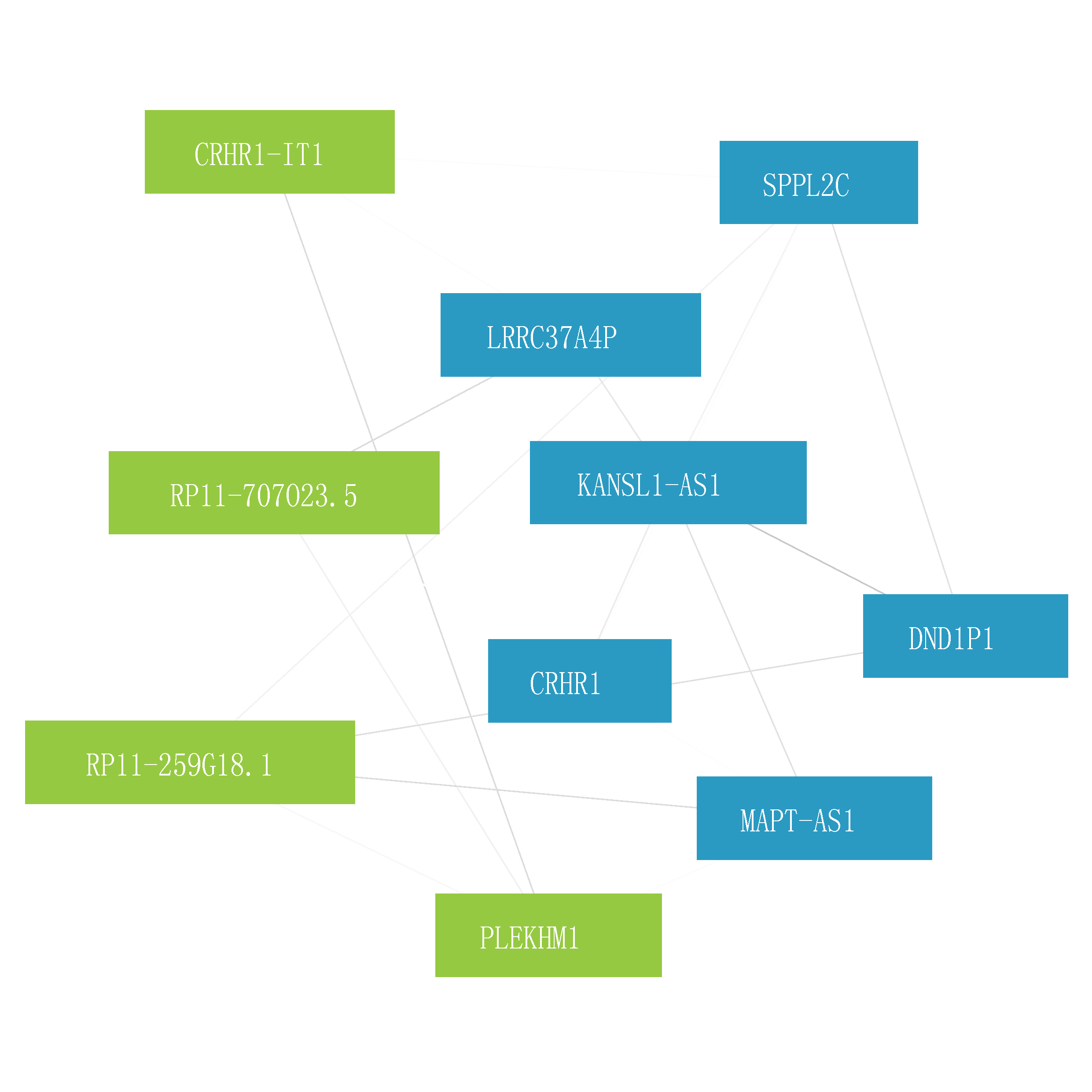


**Figure S14. Co-expression network of 10 transcriptome-wide significant genes (in both PsychENCODE and GTEx datasets) of anxiety.** Different colors indicate different clusters.


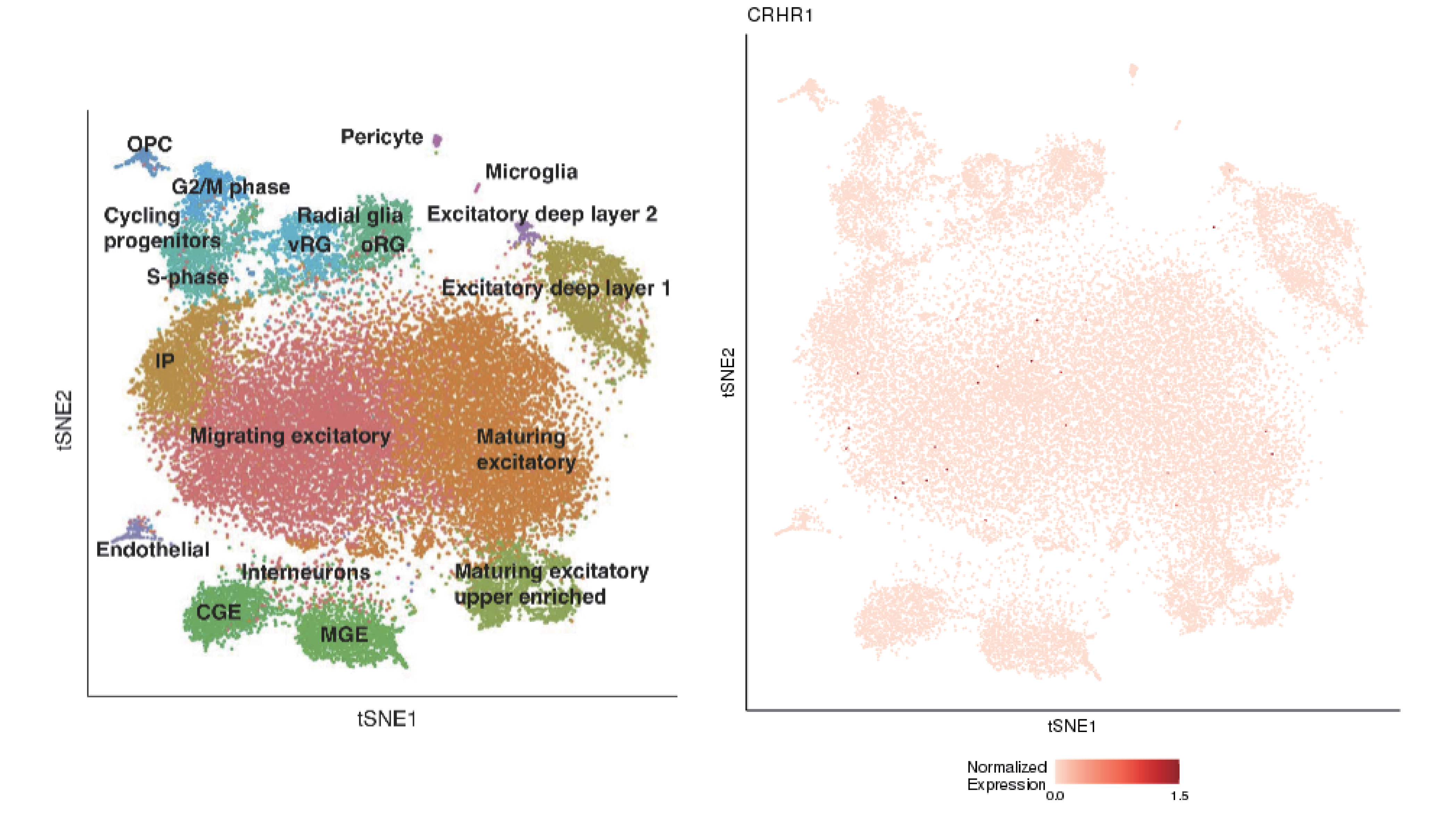


**Figure S15. *CRHR1* gene expression in single cell dataset of developing human neocortex (**[**http://solo.bmap.ucla.edu/shiny/webapp/**](http://solo.bmap.ucla.edu/shiny/webapp/)**)**


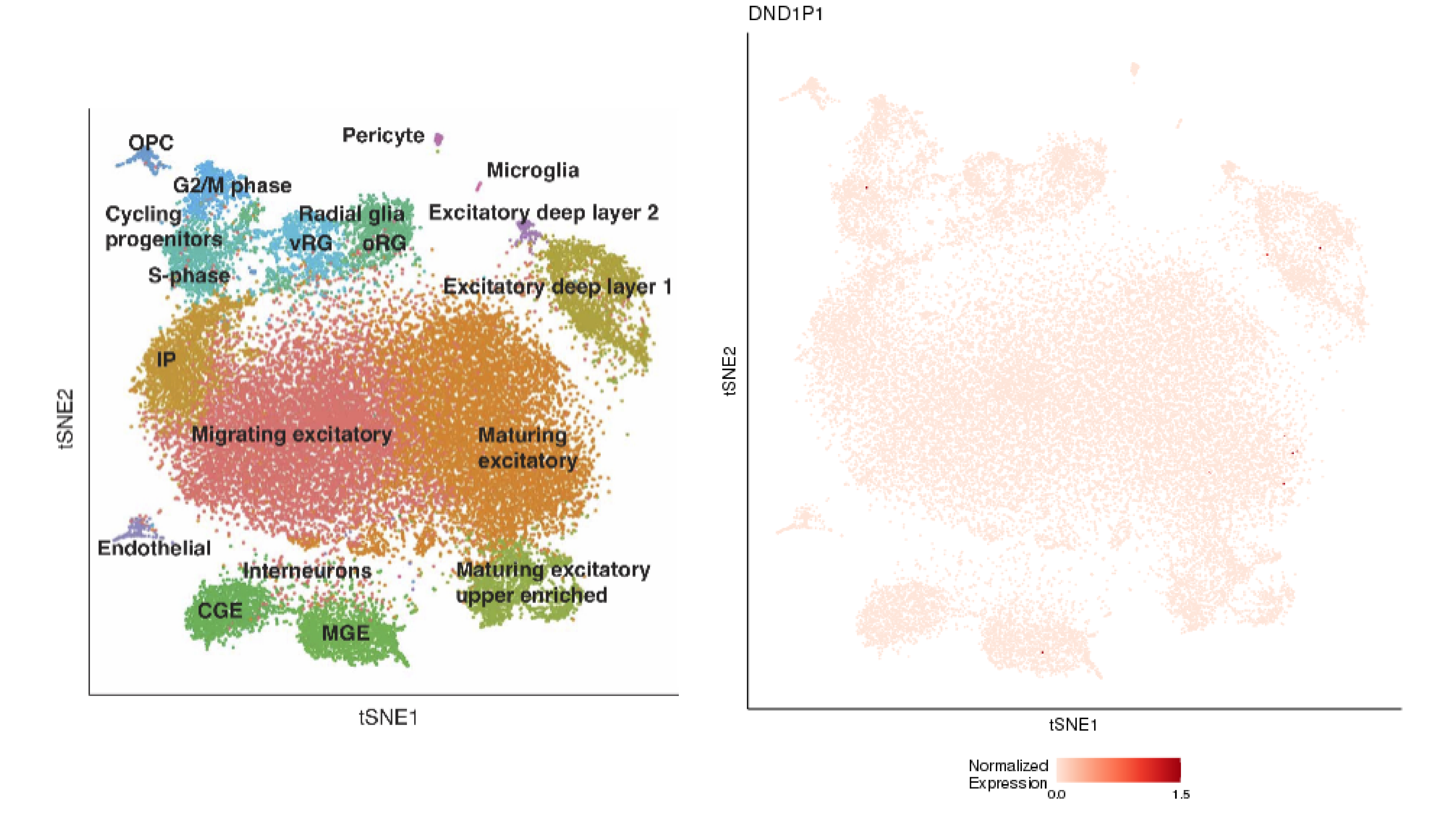


**Figure S16. *DND1P1* gene expression in single cell dataset of developing human neocortex (**[**http://solo.bmap.ucla.edu/shiny/webapp/**](http://solo.bmap.ucla.edu/shiny/webapp/)**)**


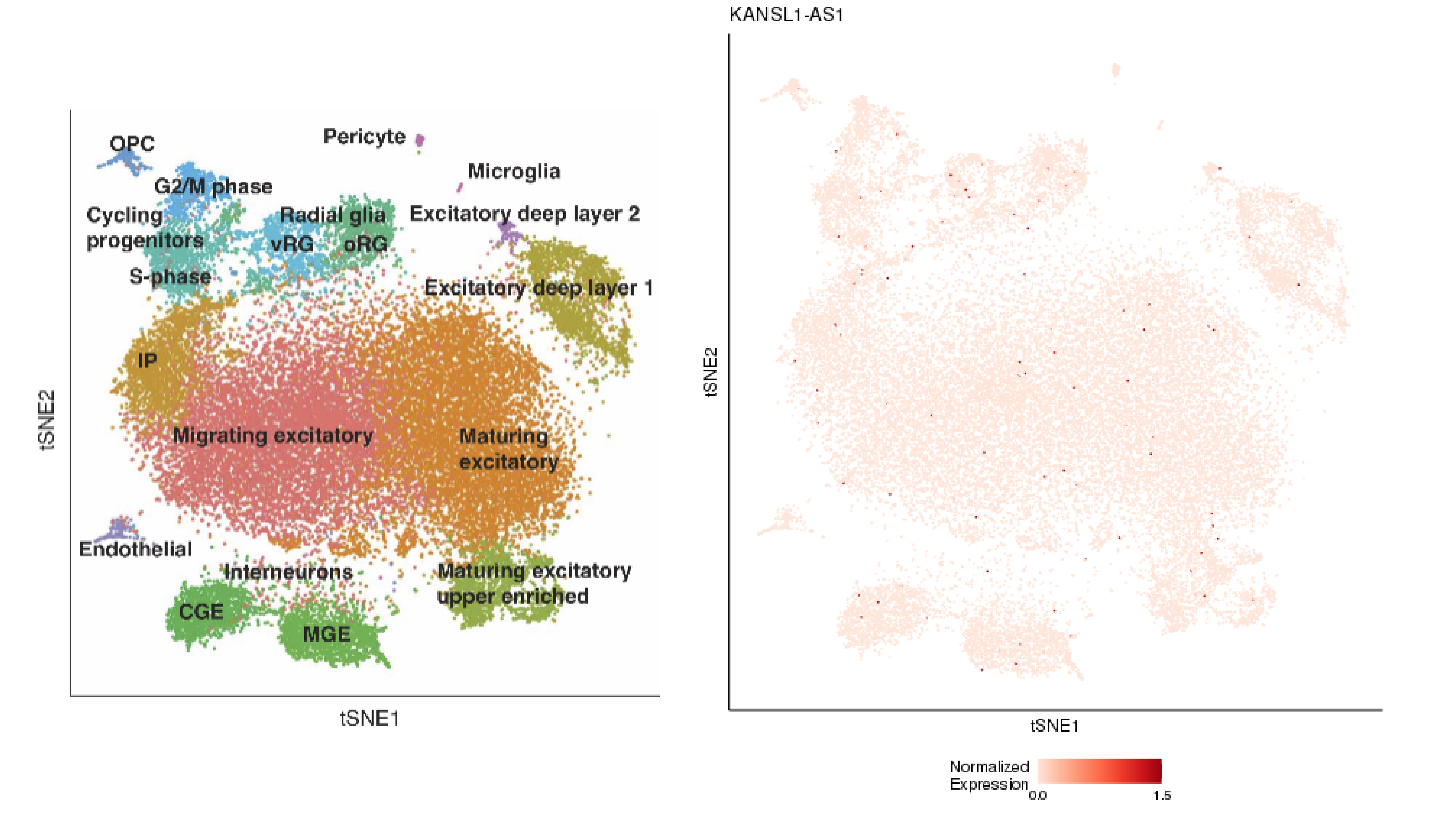


**Figure S17. *KANSL1-AS1* gene expression in single cell dataset of developing human neocortex (**[**http://solo.bmap.ucla.edu/shiny/webapp/**](http://solo.bmap.ucla.edu/shiny/webapp/)**)**


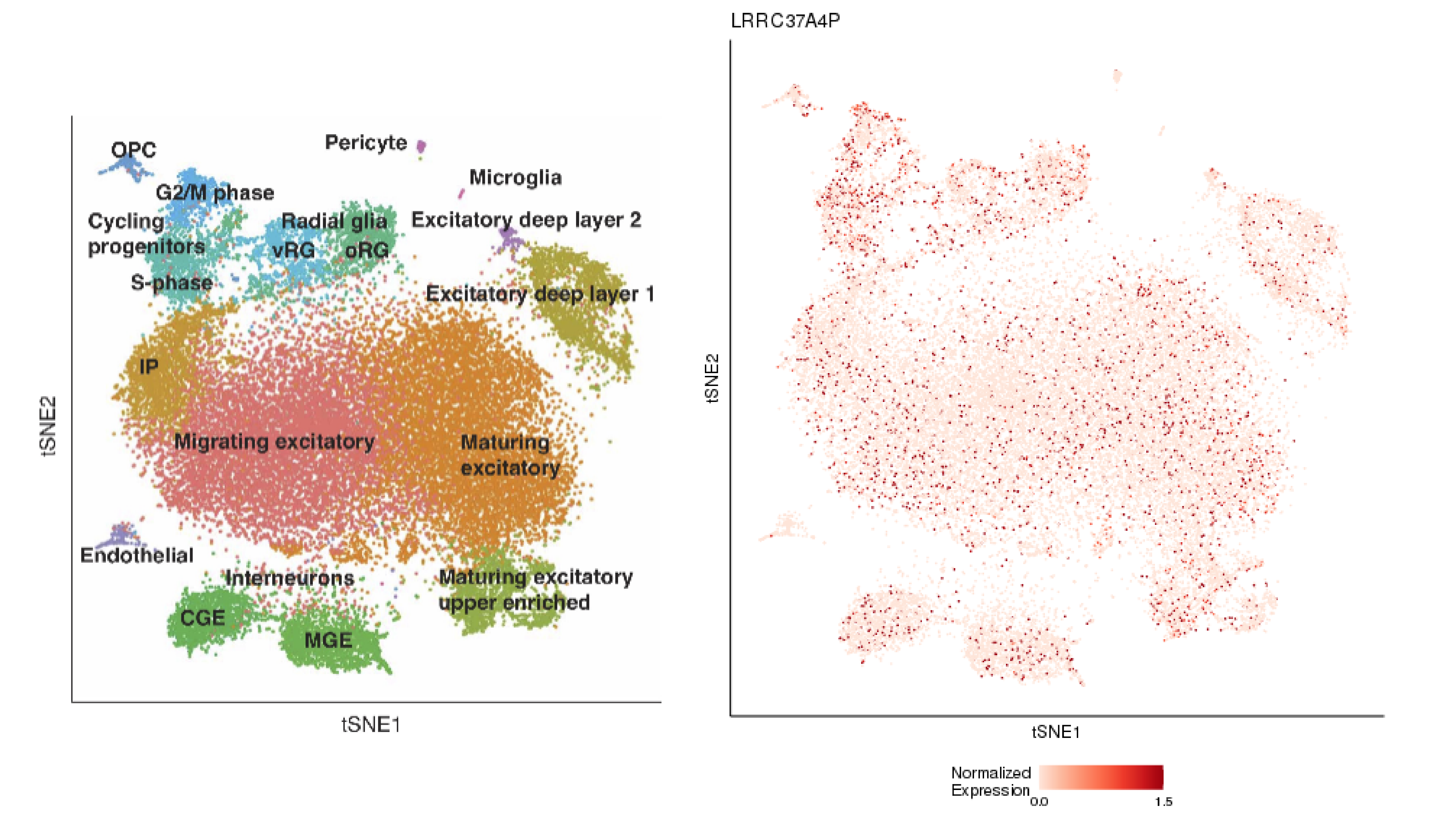


**Figure S18. *LRRC37A4P* gene expression in single cell dataset of developing human neocortex (**[**http://solo.bmap.ucla.edu/shiny/webapp/**](http://solo.bmap.ucla.edu/shiny/webapp/)**)**


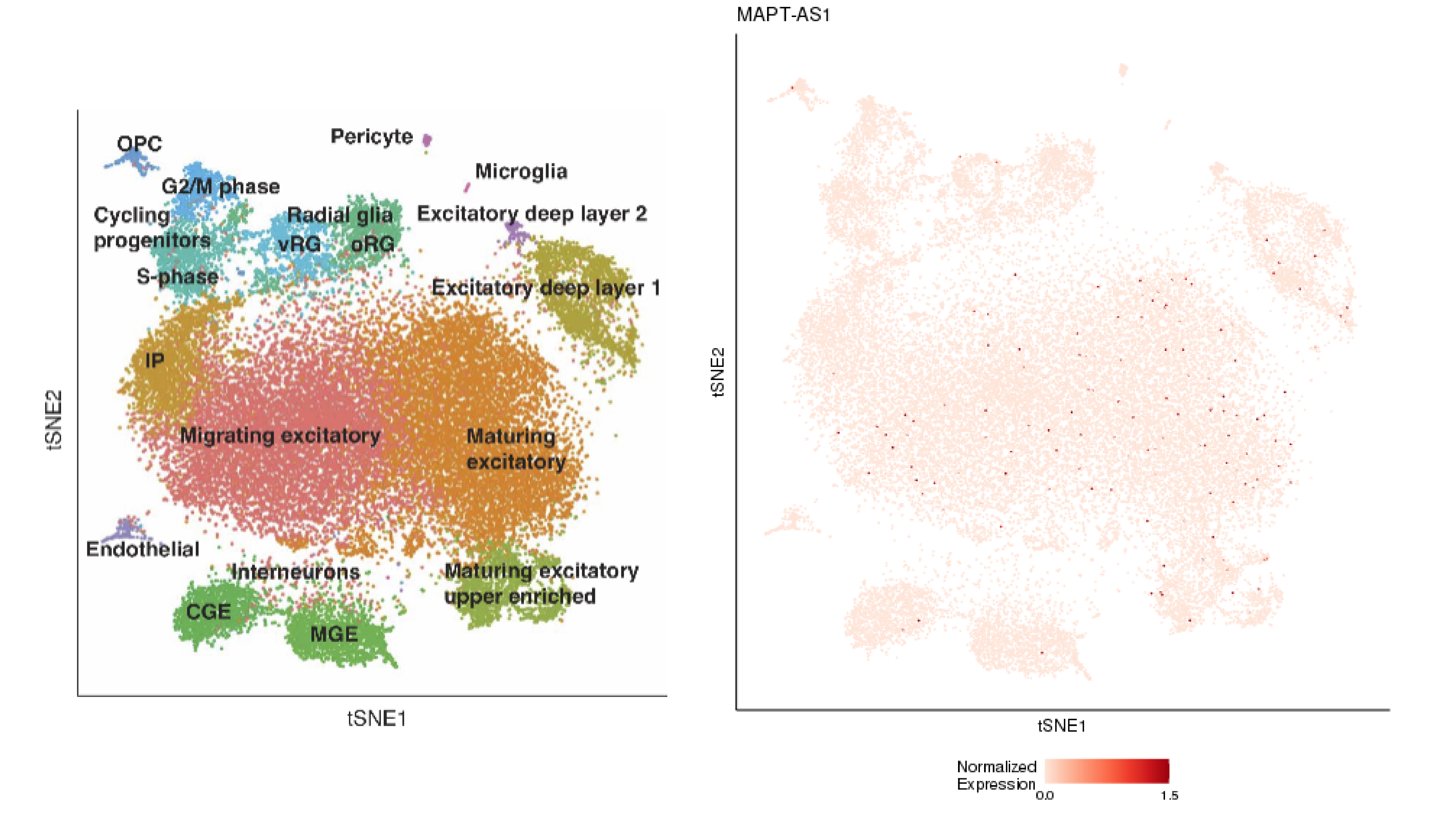


**Figure S19. *MAPT-AS1* gene expression in single cell dataset of developing human neocortex (**[**http://solo.bmap.ucla.edu/shiny/webapp/**](http://solo.bmap.ucla.edu/shiny/webapp/)**)**


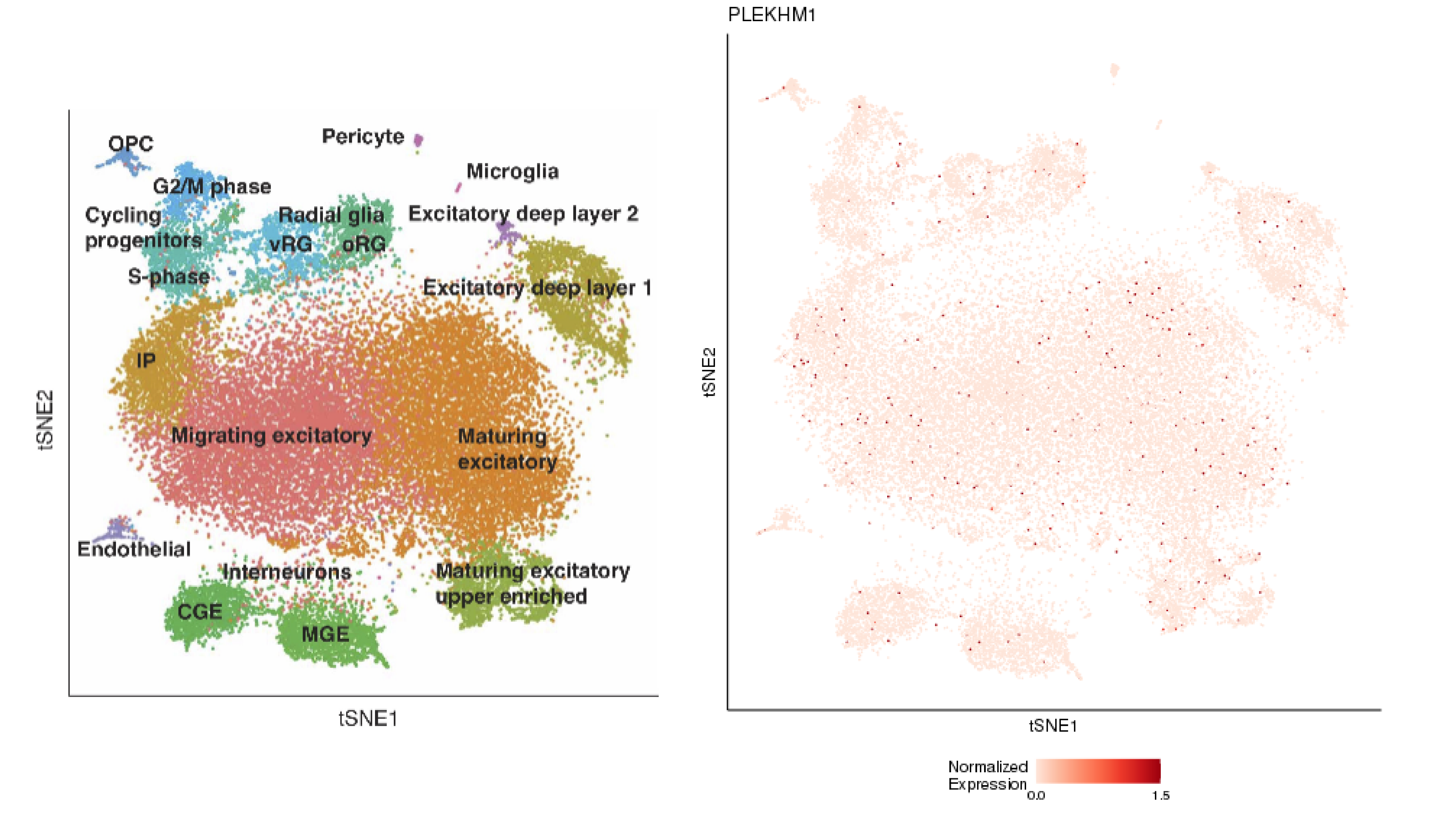


**Figure S20. *PLEKHM1* gene expression in single cell dataset of developing human neocortex (**[**http://solo.bmap.ucla.edu/shiny/webapp/**](http://solo.bmap.ucla.edu/shiny/webapp/)**)**

**Table S1. Transcriptome-wide significant genes (brain SNP-gene expression weights were from PsychENCODE ).**

| **Gene** | **Chr** | **TWAS.model** | **TWAS.P** | **TWAS.panel** |
| --- | --- | --- | --- | --- |
| *KANSL1-AS1* | 17 | enet | 6.28E-08 | PsychENCODE |
| *GLYCTK* | 3 | lasso | 1.17E-07 | PsychENCODE |
| *RN7SL199P* | 17 | bslmm | 2.02E-07 | PsychENCODE |
| *CRHR1* | 17 | lasso | 3.44E-07 | PsychENCODE |
| *RN7SL656P* | 17 | enet | 3.47E-07 | PsychENCODE |
| *RP11-798G7.7* | 17 | lasso | 3.52E-07 | PsychENCODE |
| *CRHR1-IT1* | 17 | enet | 4.27E-07 | PsychENCODE |
| *MAPT* | 17 | enet | 4.72E-07 | PsychENCODE |
| *SPPL2C* | 17 | blup | 5.29E-07 | PsychENCODE |
| *STH* | 17 | blup | 6.40E-07 | PsychENCODE |
| *RP11-707O23.5* | 17 | enet | 7.71E-07 | PsychENCODE |
| *LRRC37A* | 17 | enet | 9.75E-07 | PsychENCODE |
| *RP11-259G18.1* | 17 | lasso | 1.05E-06 | PsychENCODE |
| *MAPT-AS1* | 17 | lasso | 1.23E-06 | PsychENCODE |
| *LRRC37A4P* | 17 | enet | 1.56E-06 | PsychENCODE |
| *PLEKHM1* | 17 | bslmm | 1.62E-06 | PsychENCODE |
| *RNF123* | 3 | enet | 1.66E-06 | PsychENCODE |
| *DND1P1* | 17 | lasso | 3.16E-06 | PsychENCODE |
| *KHK* | 2 | lasso | 3.35E-06 | PsychENCODE |

**Table S2. Transcriptome-wide significant genes (brain SNP-gene expression weights were from GTEx).**

| **Gene** | **Chromosome** | **TWAS.P(model)** | **brain_region** |
| --- | --- | --- | --- |

| *AMT* | 3 | 1.08E-06(blup) | Hypothalamus |
| --- | --- | --- | --- |
| *ARHGAP27* | 17 | 6.18E-07(lasso) | Nucleus_accumbens_basal_ganglia |
| *CRHR1* | 17 | 9.11E-07(lasso) | Putamen |
| *CRHR1-IT1* | 17 | 1.72E-07(lasso) | Nucleus_accumbens_basal_ganglia |
| *CRHR1-IT1* | 17 | 3.66E-07(enet) | Hippocampus |
| *CRHR1-IT1* | 17 | 5.54E-07(lasso) | Anterior_cingulate_cortex_BA24 |
| *CRHR1-IT1* | 17 | 8.51E-07(lasso) | Spinal_cord_cervical |
| *CRHR1-IT1* | 17 | 8.56E-07(lasso) | Putamen |
| *CRHR1-IT1* | 17 | 8.87E-07(lasso) | Frontal_Cortex_BA9 |
| *CRHR1-IT1* | 17 | 1.00E-06(lasso) | Caudate_basal_ganglia |
| *CRHR1-IT1* | 17 | 1.01E-06(enet) | Hypothalamus |
| *CRHR1-IT1* | 17 | 2.03E-06(lasso) | Substantia_nigra |
| *DND1P1* | 17 | 3.44E-07(enet) | Anterior_cingulate_cortex_BA24 |
| *DND1P1* | 17 | 3.60E-07(enet) | Frontal_Cortex_BA9 |
| *DND1P1* | 17 | 6.99E-07(lasso) | Cortex |
| *DND1P1* | 17 | 8.12E-07(lasso) | Putamen |
| *DND1P1* | 17 | 8.79E-07(lasso) | Cerebellar_Hemisphere |
| *DND1P1* | 17 | 8.97E-07(lasso) | Nucleus_accumbens_basal_ganglia |
| *DND1P1* | 17 | 9.41E-07(lasso) | Amygdala |
| *DND1P1* | 17 | 1.02E-06(lasso) | Hypothalamus |
| *DND1P1* | 17 | 1.07E-06(lasso) | Caudate_basal_ganglia |
| *DND1P1* | 17 | 1.38E-06(lasso) | Hippocampus |
| *DND1P1* | 17 | 1.70E-06(lasso) | Spinal_cord_cervical |
| *KANSL1-AS1* | 17 | 9.87E-08(enet) | Hypothalamus |
| *KANSL1-AS1* | 17 | 1.67E-07(lasso) | Substantia_nigra |
| *KANSL1-AS1* | 17 | 4.72E-07(lasso) | Cerebellum |
| *KANSL1-AS1* | 17 | 5.10E-07(enet) | Nucleus_accumbens_basal_ganglia |
| *KANSL1-AS1* | 17 | 5.63E-07(lasso) | Anterior_cingulate_cortex_BA24 |
| *KANSL1-AS1* | 17 | 5.84E-07(enet) | Cortex |
| *KANSL1-AS1* | 17 | 6.96E-07(lasso) | Cerebellar_Hemisphere |
| *KANSL1-AS1* | 17 | 8.53E-07(lasso) | Caudate_basal_ganglia |
| *KANSL1-AS1* | 17 | 8.60E-07(lasso) | Hippocampus |
| *KANSL1-AS1* | 17 | 8.79E-07(lasso) | Putamen |
| *KANSL1-AS1* | 17 | 8.81E-07(lasso) | Frontal_Cortex_BA9 |
| *KANSL1-AS1* | 17 | 9.49E-07(lasso) | Spinal_cord_cervical |
| *LRRC37A4P* | 17 | 1.53E-07(lasso) | Substantia_nigra |
| *LRRC37A4P* | 17 | 3.30E-07(lasso) | Hypothalamus |
| *LRRC37A4P* | 17 | 6.24E-07(enet) | Amygdala |
| *LRRC37A4P* | 17 | 6.54E-07(lasso) | Caudate_basal_ganglia |
| *LRRC37A4P* | 17 | 8.79E-07(lasso) | Nucleus_accumbens_basal_ganglia |
| *LRRC37A4P* | 17 | 9.45E-07(enet) | Hippocampus |
| *LRRC37A4P* | 17 | 1.01E-06(enet) | Anterior_cingulate_cortex_BA24 |
| *LRRC37A4P* | 17 | 1.04E-06(lasso) | Spinal_cord_cervical |
| *MAPT-AS1* | 17 | 4.93E-07(enet) | Cerebellar_Hemisphere |
| *PLEKHM1* | 17 | 6.89E-07(enet) | Cerebellar_Hemisphere |
| *PLEKHM1* | 17 | 9.69E-07(lasso) | Cortex |
| *RGS19* | 20 | 1.61E-08(lasso) | Cerebellum |
| *RP11-259G18.1* | 17 | 8.71E-07(lasso) | Caudate_basal_ganglia |
| *RP11-259G18.1* | 17 | 8.92E-07(lasso) | Hippocampus |
| *RP11-259G18.1* | 17 | 2.39E-06(blup) | Substantia_nigra |
| *RP11-259G18.2* | 17 | 1.09E-06(lasso) | Anterior_cingulate_cortex_BA24 |
| *RP11-259G18.2* | 17 | 1.15E-06(lasso) | Hippocampus |
| *RP11-259G18.2* | 17 | 1.32E-06(blup) | Amygdala |
| *RP11-259G18.2* | 17 | 2.28E-06(lasso) | Substantia_nigra |
| *RP11-259G18.3* | 17 | 2.74E-07(enet) | Cerebellum |
| *RP11-707O23.5* | 17 | 7.65E-07(lasso) | Spinal_cord_cervical |
| *RP11-707O23.5* | 17 | 7.84E-07(lasso) | Substantia_nigra |
| *RP11-707O23.5* | 17 | 8.17E-07(lasso) | Putamen |
| *RP11-707O23.5* | 17 | 8.37E-07(lasso) | Frontal_Cortex_BA9 |
| *RP11-707O23.5* | 17 | 8.72E-07(lasso) | Hypothalamus |
| *RP11-707O23.5* | 17 | 8.89E-07(lasso) | Nucleus_accumbens_basal_ganglia |
| *RP11-707O23.5* | 17 | 9.11E-07(lasso) | Anterior_cingulate_cortex_BA24 |
| *RP11-707O23.5* | 17 | 9.43E-07(enet) | Cortex |
| *RP11-707O23.5* | 17 | 9.47E-07(lasso) | Hippocampus |
| *RP11-707O23.5* | 17 | 1.48E-06(lasso) | Amygdala |
| *RP11-798G7.5* | 17 | 2.22E-07(lasso) | Cerebellar_Hemisphere |
| *RP11-798G7.8* | 17 | 3.77E-07(lasso) | Cerebellar_Hemisphere |
| *SPPL2C* | 17 | 1.05E-07(blup) | Cerebellar_Hemisphere |
| *SPPL2C* | 17 | 6.12E-07(enet) | Frontal_Cortex_BA9 |

|  |  |  |  |
| --- | --- | --- | --- |

**Table S3. The MAGMA GTEx tissue enrichment analysis.**

| **GTEx tissues** | **P** | **FDR** |
| --- | --- | --- |
| Brain_Cortex | 0.0025778 | 0.0549594 |
| Brain_Frontal_Cortex_BA9 | 0.0030533 | 0.0549594 |
| Testis | 0.0021316 | 0.0549594 |
| Brain_Anterior_cingulate_cortex_BA24 | 0.012848 | 0.173448 |
| Brain_Cerebellar_Hemisphere | 0.019293 | 0.173637 |
| Brain_Cerebellum | 0.019268 | 0.173637 |
| Pituitary | 0.032674 | 0.252056571 |
| Brain_Nucleus_accumbens_basal_ganglia | 0.045646 | 0.3081105 |
| Brain_Hypothalamus | 0.063277 | 0.379662 |
| Brain_Caudate_basal_ganglia | 0.071289 | 0.3849606 |
| Brain_Amygdala | 0.088304 | 0.397368 |
| Brain_Hippocampus | 0.086969 | 0.397368 |
| Brain_Putamen_basal_ganglia | 0.10515 | 0.436776923 |
| Adipose_Subcutaneous | 0.9437 | 0.993630566 |
| Adipose_Visceral_Omentum | 0.96784 | 0.993630566 |
| Adrenal_Gland | 0.68118 | 0.993630566 |
| Artery_Aorta | 0.85469 | 0.993630566 |
| Artery_Coronary | 0.92939 | 0.993630566 |
| Artery_Tibial | 0.82041 | 0.993630566 |
| Bladder | 0.78372 | 0.993630566 |
| Brain_Spinal_cord_cervical_c-1 | 0.55159 | 0.993630566 |
| Brain_Substantia_nigra | 0.26443 | 0.993630566 |
| Breast_Mammary_Tissue | 0.88913 | 0.993630566 |
| Cells_Cultured_fibroblasts | 0.82867 | 0.993630566 |
| Cells_EBV-transformed_lymphocytes | 0.74206 | 0.993630566 |
| Cervix_Ectocervix | 0.78268 | 0.993630566 |
| Cervix_Endocervix | 0.60108 | 0.993630566 |
| Colon_Sigmoid | 0.51672 | 0.993630566 |
| Colon_Transverse | 0.65875 | 0.993630566 |
| Esophagus_Gastroesophageal_Junction | 0.59723 | 0.993630566 |
| Esophagus_Mucosa | 0.94377 | 0.993630566 |
| Esophagus_Muscularis | 0.58612 | 0.993630566 |
| Fallopian_Tube | 0.47663 | 0.993630566 |
| Heart_Atrial_Appendage | 0.59112 | 0.993630566 |
| Heart_Left_Ventricle | 0.57579 | 0.993630566 |
| Kidney_Cortex | 0.97181 | 0.993630566 |
| Kidney_Medulla | 0.97523 | 0.993630566 |
| Liver | 0.97178 | 0.993630566 |
| Minor_Salivary_Gland | 0.93126 | 0.993630566 |
| Muscle_Skeletal | 0.81025 | 0.993630566 |
| Nerve_Tibial | 0.31926 | 0.993630566 |
| Ovary | 0.4015 | 0.993630566 |
| Pancreas | 0.91028 | 0.993630566 |
| Prostate | 0.69603 | 0.993630566 |
| Skin_Not_Sun_Exposed_Suprapubic | 0.67618 | 0.993630566 |
| Skin_Sun_Exposed_Lower_leg | 0.718 | 0.993630566 |
| Small_Intestine_Terminal_Ileum | 0.77918 | 0.993630566 |
| Spleen | 0.68956 | 0.993630566 |
| Stomach | 0.94608 | 0.993630566 |
| Thyroid | 0.73999 | 0.993630566 |
| Uterus | 0.32584 | 0.993630566 |
| Vagina | 0.77738 | 0.993630566 |
| Whole_Blood | 0.85616 | 0.993630566 |
| Lung | 0.9968 | 0.9968 |

**Table S4. The MAGMA single cells enrichment analysis.**

| **Single cell groups** | **P** | **FDR** |
| --- | --- | --- |
| Hindbrain.neurons | 0.00018441 | 0.00719199 |
| Di..and.mesencephalon.inhibitory.neurons | 0.0012299 | 0.02398305 |
| Di..and.mesencephalon.excitatory.neurons | 0.014452 | 0.187876 |
| Cholinergic.and.monoaminergic.neurons | 0.03262 | 0.318045 |
| Enteric.neurons | 0.049889 | 0.357019 |
| Olfactory.inhibitory.neurons | 0.054926 | 0.357019 |
| Cerebellum.neurons | 0.10868 | 0.529815 |
| Telencephalon.inhibitory.interneurons | 0.10356 | 0.529815 |
| Peripheral.sensory.neurofilament.neurons | 0.1405 | 0.608833333 |
| Enteric.glia | 0.19361 | 0.6372 |
| Spinal.cord.excitatory.neurons | 0.1645 | 0.6372 |
| Spinal.cord.inhibitory.neurons | 0.20138 | 0.6372 |
| Telencephalon.projecting.excitatory.neurons | 0.2124 | 0.6372 |
| Vascular.and.leptomeningeal.cells | 0.25424 | 0.70824 |
| Choroid.epithelial.cells | 0.43239 | 0.733183043 |
| Dentate.gyrus.granule.neurons | 0.31948 | 0.733183043 |
| Glutamatergic.neuroblasts | 0.33712 | 0.733183043 |
| Non.glutamatergic.neuroblasts | 0.34166 | 0.733183043 |
| Pericytes | 0.39219 | 0.733183043 |
| Schwann.cells | 0.39587 | 0.733183043 |
| Telencephalon.projecting.inhibitory.neurons | 0.29023 | 0.733183043 |
| Vascular.endothelial.cells | 0.3746 | 0.733183043 |
| Vascular.smooth.muscle.cells | 0.4232 | 0.733183043 |
| Oligodendrocytes | 0.52744 | 0.806418889 |
| Peripheral.sensory.non.peptidergic.neurons | 0.54755 | 0.806418889 |
| Perivascular.macrophages | 0.55829 | 0.806418889 |
| Subcommissural.organ.hypendymal.cells | 0.54633 | 0.806418889 |
| Olfactory.ensheathing.cells | 0.60352 | 0.81764129 |
| Peptidergic.neurons | 0.64992 | 0.81764129 |
| Peripheral.sensory.peptidergic.neurons | 0.62649 | 0.81764129 |
| Sympathetic.cholinergic.neurons | 0.63986 | 0.81764129 |
| Dentate.gyrus.radial.glia.like.cells | 0.67702 | 0.817983636 |
| Subventricular.zone.radial.glia.like.cells | 0.69214 | 0.817983636 |
| Astrocytes | 0.79014 | 0.890882571 |
| Sympathetic.noradrenergic.neurons | 0.79951 | 0.890882571 |
| Ependymal.cells | 0.87905 | 0.902182895 |
| Microglia | 0.8726 | 0.902182895 |
| Oligodendrocyte.precursor.cells | 0.86559 | 0.902182895 |
| Satellite.glia | 0.931 | 0.931 |
